# TRAF3 activates STING-mediated suppression of EV-A71 and target of viral evasion

**DOI:** 10.1038/s41392-022-01287-2

**Published:** 2023-02-24

**Authors:** Wenwen Zheng, Zhenbang Zhou, Yajuan Rui, Runxin Ye, Fengyan Xia, Fei Guo, Xiaoman Liu, Jiaming Su, Meng Lou, Xiao-Fang Yu

**Affiliations:** 1 Cancer Institute (Key Laboratory of Cancer Prevention and Intervention, China National Ministry of Education), The Second Affiliated Hospital, Zhejiang University School of Medicine, Hangzhou, Zhejiang 310009 China; 2 National Health Commission of the People’s Republic of China Key Laboratory of Systems Biology of Pathogens, Institute of Pathogen Biology and Center for AIDS Research, Chinese Academy of Medical Sciences and Peking Union Medical College, Beijing, 100730 China; 3 Cancer Center, Zhejiang University, Hangzhou, Zhejiang 310058 China

**Keywords:** Innate immunity, Infectious diseases, Infection

## Abstract

Innate immunity represents one of the main host responses to viral infection.^1–3^ STING (Stimulator of interferon genes), a crucial immune adapter functioning in host cells, mediates cGAS (Cyclic GMP-AMP Synthase) sensing of exogenous and endogenous DNA fragments and generates innate immune responses.^4^ Whether STING activation was involved in infection and replication of enterovirus remains largely unknown. In the present study, we discovered that human enterovirus A71 (EV-A71) infection triggered STING activation in a cGAS dependent manner. EV-A71 infection caused mitochondrial damage and the discharge of mitochondrial DNA into the cytosol of infected cells. However, during EV-A71 infection, cGAS-STING activation was attenuated. EV-A71 proteins were screened and the viral protease 2A^pro^ had the greatest capacity to inhibit cGAS-STING activation. We identified TRAF3 as an important factor during STING activation and as a target of 2A^pro^. Supplement of TRAF3 rescued cGAS-STING activation suppression by 2A^pro^. TRAF3 supported STING activation mediated TBK1 phosphorylation. Moreover, we found that 2A^pro^ protease activity was essential for inhibiting STING activation. Furthermore, EV-D68 and CV-A16 infection also triggered STING activation. The viral protease 2A^pro^ from EV-D68 and CV-A16 also had the ability to inhibit STING activation. As STING activation prior to EV-A71 infection generated cellular resistance to EV-A71 replication, blocking EV-A71-mediated STING suppression represents a new anti-viral target.

## Introduction

EV-A71 is a member of the genus Enterovirus in the *Picornaviridae* family which mainly contributed to pathogenesis of hand-foot-and-mouth disease (HFMD). HFMD is a disease that usually affects young children and is mild by most people but possibly be related to a severe form of brainstem encephalitis, pulmonary edema, and is occasionally fatal.^5–7^ EV-A71 is a non-enveloped virus bearing a capsid with icosahedral symmetry containing a non-segmented, single-stranded, positive-sense genomic RNA that is about 7,500 nucleotides long with an open reading frame (ORF) flanked by untranslated regions (UTRs) at the 5' and 3' termini.^8^ Genomic RNA is served as the template for translation and synthesis of negative-strand viral RNA. Translation of the genomic RNA is driven by an internal ribosome entry site (IRES) located in the 5' UTR; however, it is not rested with the eukaryotic cap structure and is instead processed by the viral 2 A protease (2A^pro^) and 3 C protease cleavage to generate 11 viral proteins. Genome replication depends on the negative strands synthesized by RNA-dependent RNA polymerase 3D.^9–11^

Host innate immune responses play significant roles in defenses against EV-A71 infection. It reacts promptly to the invasion of pathogens and usually effectively limit infections, subsequently prime signals that prime adaptive immune responses for removal of infectious organisms.^12–14^ There are ten members of the toll-like receptor (TLR) family in humans, and TLR3 is a key sensor of viral genome RNA (dsRNA) during EV-A71 infection.^15^ TLR3 activation triggers downstream signaling for type I IFN induction and anti-viral responses.^16–18^ RIG-I-like receptor (RLR) pathways include retinoic acid inducible gene I (RIG-I) and melanoma differentiation-associated protein 5 (MDA5), both of which serve as guards in the cytoplasm for detecting RNA virus infection.^19,20^ MDA5 recognizes long dsRNA or RNA missing 2-O-methylation at the 5 ends.^21,22^ RIG-I recognizes short dsRNA or viral RNA species containing 5' triphosphates or diphosphates.^23,24^ After binding target RNA, MDA5 and RIG-I activate and recruit the mitochondrial adapter molecule (MAVS) to prime innate immune responses.

For EV-A71 specifically, various studies have implicated MDA5 as a sensor of EV-A71 infection.^25–27^ Furthermore, it has been shown that arrestin domain-containing 4 interacts with MDA5 to facilitate its ubiquitination and activation to generate pro-inflammatory cytokines during EV-A71 infection.^28^ Interestingly, MDA5 polymorphisms have been reported to associate with HFMD severity in children.^29^ Additionally, EV-A71 has evolved various counter mechanisms to take the edge off host innate immune responses, these counteractions ultimately lead to efficient viral replication and enhancive host susceptibility to the disease.^12,30^ It has been reported by a lot of relevant research groups that EV-A71 infection could not trigger RLR mediated innate immune responses in HEK-293T,^31^ HeLa^25,32,33^ and RD^31^ cells. Interestingly, naked EV-A71 RNA triggered RLR pathway activation in HeLa cells^25^ comparing to native infection, suggesting that EV-A71 particles have mechanisms for RLR evasion.^31,32^

In addition to sensing viral RNA, cGAS-STING acts as a crucial sensor in sensing viral DNA during multiple DNA viruses infection and other abnormal DNA fragment accumulation during disease.^34,35^ The activator of STING—2'3'-cyclic GMP-AMP (cGAMP) is produced by the DNA pattern classical recognition receptor cyclic GMP-AMP synthase (cGAS), which recognizes foreign DNA such as pathogens or small DNA fragments in cells.^4,36^ Endogenous source of small DNA fragments is reported mainly from different type of DNA damage, including mitochondrial damage and subsequent mitochondrial DNA (mtDNA)release,^37^ and the formation of micronuclei,^38^ et al. 2'3'-cGAMP can be directly produced in the cytoplasm of DNA-producing cells or enter the cytoplasm through virus-carrying,^4,39^ cell membrane channels, and cell membrane transporters, including SLC19A1, LRRC8, and P2X7R,^40–42^ et al. 2'3'-cGAMP then activates STING to form a complex with endogenous TANK-binding kinase 1 (TBK1)-Interferon Regulatory Factor 3 (IRF3) to activate host interferon gene transcription.^36^ Except for cGAS dependent STING activation, cGAS independent activation has also been reported. For example, influenza A virus can interact with STING and prime its activation.^43^ The classic output of cGAS-STING activation is IRF activation and type I interferon response. Meanwhile, nuclear factor kappa-B (NF-кB) is another important STING activated pathway. As one of the significant pathways of the human innate immune response, NF-кB activate the transcription and expression of a variety of antiviral components induced by innate immunity, including a variety of type I interferons, interleukins, inflammatory factors,^44,45^ and can induce dendritic cell maturation and antigen presentation.^46^

However, it is not clear whether EV-A71 infection primes the innate immune response through the STING-mediated signaling pathway and the underlying mechanisms. In the present study, STING activation triggered by EV-A71 infection was observed. This process was dependent on cGAS activity of 2'3'-cGAMP production and continuous EV-A71 replication in host cells. Moreover, EV-A71 developed a strategy to suppress cGAS-STING function. EV-A71 2A^pro^ strongly suppressed STING activation through its cleavage of TRAF3 which is important factor for STING triggered innate immune activation. TRAF3 turn out to be crucial for STING-TBK1 interaction and downstream TBK1 phosphorylation. In particular, 2A^pro^ has a potent ability to inactivate STING-mediated signaling and thus represents a new viral target for therapeutic intervention.

## Results

### EV-A71 infection triggers STING mediated immune activation and viral suppression

**Fig. 1:**
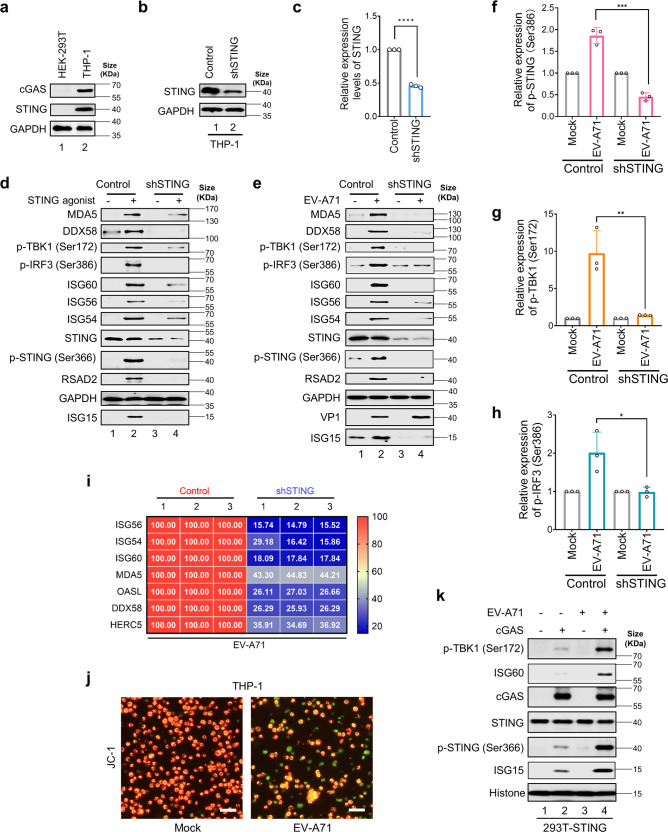
EV-A71 infection triggers STING-mediated immune activation and viral suppression. **a** Relative expression of STING and cGAS in HEK-293T and THP-1 cells. Cells were cultured and harvested for western blot analysis. STING and cGAS levels were evaluated using anti-STING and anti-cGAS antibodies. GAPDH was used as a loading control. **b**, **c** STING was efficiently down-regulated in shSTING-THP-1 cells compared with control cells and was quantified using ImageJ software (National Institutes of Health, Maryland, USA). Cells were cultured and harvested for western blot analysis. STING was tested using an anti-STING antibody. GAPDH was used as a loading control. **d** STING could be activated in control THP-1 cells and such activation was impaired when STING was down-regulated. Equal numbers of THP-1 control or shSTING cells were seeded into 12-well plates and treated with 100 nM STING agonist for 12 h and harvested for western blot analysis. MDA5, DDX58, p-TBK1 (Ser172), p-IRF3 (Ser386), ISG60, ISG56, ISG54, STING, p-STING (Ser366), RSAD2, ISG15 and GAPDH were probed using the indicated antibodies. GAPDH was used as a loading control. **e**–**h** EV-A71 infection could efficiently induce STING-triggered down-stream gene activation. Equal volumes of EV-A71 (MOI of 0.5) were used to infect control/shSTING-THP-1 cells (2 × 10^5^/well in 24-well plates). Viral preservation solution was used as a negative control. 24 h later, cells were harvested for western blot analysis. MDA5, DDX58, p-TBK1 (Ser172), p-IRF3 (Ser386), ISG60, ISG56, ISG54, STING, p-STING (Ser366), RSAD2, ISG15, VP1 and GAPDH were probed using the indicated antibodies. GAPDH was used as a loading control. p-STING (Ser366) (**f**), p-TBK1 (Ser172) (**g**), and p-IRF3 (Ser386) (**h**) protein expressions were represented by western blot bands and were quantified using ImageJ software. *n* = 3 independent experiments were performed (representative immunoblots are shown). **i** Inhibition of EV-A71-stimulated human genes by silencing STING expression. THP-1 control or shSTING cells were infected with EV-A71 for 24 h and harvested for total RNA extraction, first strand cDNA synthesis, and RT-qPCR analysis with indicated interferon stimulated genes specific primers. Human genes activated by EV-A71 alone served as a positive control and were set to 100%. Total RNA was prepared from THP-1 cells and analyzed for the transcriptional level of the indicated genes by RT-qPCR (*n* = 3 independent biological experiments). Statistical significance was determined by two-sided unpaired *t*-test. **j** Representative images of JC-1-stained THP-1 cells. Cells were infected with EV-A71 for 24 h and subjected to mitochondrial membrane potential using JC-1 staining. Scale bar: 80 µm. **k** HEK-293T STING stable cell line were transfected with cGAS expression vectors or empty vectors. After 36 h, those transfected cells were then mock-infected or infected with EV-A71 at an MOI of 0.5 for 4 h and harvested for western blot analysis. p-TBK1 (Ser172), ISG60, cGAS, STING, p-STING (Ser366), ISG15, and Histone H3 were probed using the indicated antibodies. Histone H3 was used as a loading control. Data in **a**–**k** represent the average of results from three independent experiments (*n* = 3, representative immunoblots are shown). Error bars indicate the standard deviation of the data from three independent experiments. Means and standard deviations are presented. Statistical significance was determined using two-sided unpaired *t*-test, *p < 0.05; **p < 0.01; ***p < 0.001; ****p < 0.0001

In addition to sensing DNA virus infection, STING activation triggers innate immune responses during SARS-CoV-2 infection^47,48^ It is not clear whether enteroviral infections can also trigger STING activation. To address the question of whether EV-A71 infection can activate the STING pathway, the STING-positive cell line THP-1 (Fig. 1a) was used to study EV-A71 infection. STING-silenced THP-1 cells were used as controls (Figs. 1b, c). STING-silenced THP-1 cells were defective in response to STING agonist (Fig. 1d, Lanes 2 and 4) as indicated by the reduced level of STING phosphorylation at Ser366, downstream TBK1 and IRF3 phosphorylation, and suppressed production of various known interferon stimulated genes (ISGs) product such as MDA5, DDX58, ISG60, ISG56, ISG54, RSAD2, and ISG15. The reduced production of various ISG mRNA was also detected in STING-silenced THP-1 cells when compared with the control group (Supplementary Fig. 1a).

THP-1 cells could be infected by EV-A71.^49^ We therefore investigated whether EV-A71 infection could prime STING mediated immune activation. As shown in Fig. 1e, f, EV-A71 infection triggered STING activation as indicated by phosphorylation at Ser366. In addition, both TBK1 (phosphorylated at Ser172) and IRF3 (phosphorylated at Ser386) were activated in THP-1 cells after EV-A71 infection (Fig. 1e, g, and h). However, activation of TBK1 and IRF3 during EV-A71 infection was suppressed when STING was silenced (Fig. 1e, g, and h). Consistent with TBK1 and IRF3 activation, which triggers the production of type I interferons. Furthermore, MDA5, DDX58, ISG60, ISG56, ISG54, RSAD2, and ISG15 were also induced during EV-A71 infection of THP-1 cells (Fig. 1e). MDA5, DDX58, ISG60, ISG56, ISG54, RSAD2, and ISG15 induction were suppressed during EV-A71 infection in STING-silenced THP-1 cells (Fig. 1e). Furthermore, the reduced production of various ISGs and other known interferon stimulated gene mRNA was also observed in STING-silenced THP-1 cells after EV-A71 infection when compared with the control group (Fig. 1i). These results suggested that STING plays crucial roles in EV-A71 infection-triggered TBK1 and IRF3 activation and also in EV-A71 activated STING-mediated innate immune activation.

EV-A71 infection induced mitochondrial damage in THP-1 cells (Fig. 1j). EV-A71 infection also stimulated cGAMP production in THP-1 cells (Supplementary Fig. 1b) and the discharge of mtDNA into the cytosol of infected cells (Supplementary Fig. 1c). Exhausting endogenous mtDNA with dideoxycytidine (DDC)^48^ inhibited EV-A71 infection-induced cGAS-STING activation (Supplementary Fig. 1d). Blocking mtDNA release by VBIT-4, a VDAC^50^ inhibitor, suppressed EV-A71 infection induced cGAS-STING activation (Supplementary Fig. 1e).

To examine whether STING activation was initiated by EV-A71 infection directly or indirectly, we inactivated EV-A71 using β-propionolactone.^51^ We observed that inactivated EV-A71 particles did not trigger STING phosphorylation and downstream innate immune activation when compared to untreated EV-A71 (Supplementary Fig. 1f). This result indicated that productive infection by EV-A71 is required to trigger STING activation.

Furthermore, we used gain-of-function approaches to demonstrate that cGAS was required for EV-A71 infection-triggered STING activation (Fig. 1k). Notably, EV-A71 infection did not trigger RLR mediated immune activation in HEK-293T cells (Fig. 1k, line 3) as shown in previous studies.^31^ Moreover, we have also demonstrated that MAVS which is critical for RLR pathway activation (Supplementary Fig. 1g) was dispensable for EV-A71 infection-triggered STING phosphorylation (Supplementary Fig. 1h).

Above observation was further confirmed using cGAS knock-out THP-1 cells (Supplementary Fig. 2a and b). EV-A71 infection could trigger STING activation in human primary colonic mucosal epithelial cells (Supplementary Fig. 2c), human NCM460 (normal colon mucosal) cells (Supplementary Fig. 2d), and HUVEC (human umbilical vein endothelial cells) cells (Supplementary Fig. 2e).

### EV-A71 infection inhibits cGAS-STING pathway activation

**Fig. 2:**
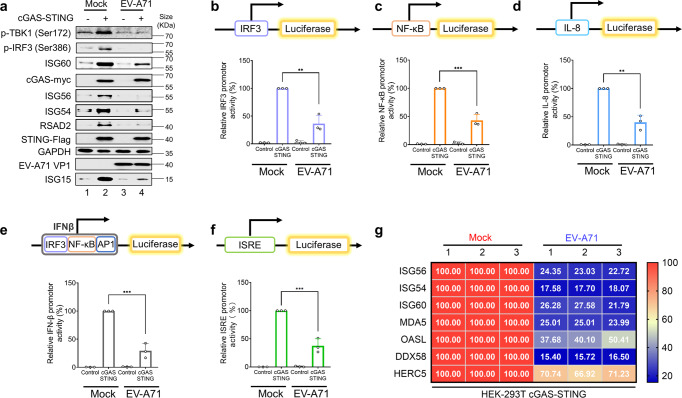
EV-A71 infection inhibits cGAS-STING pathway activation. **a** HEK-293T cells were infected with EV-A71 (MOI of 0.5) or mock-infected with virus preservation solution. Twenty-two hours later, cells were transfected with *IRF3*, *NF-κB*, *IL-8*, *IFNβ*, or *ISRE* promoter, together with cGAS-STING expression vectors or empty vector. Eighteen hours post-transfection, cells were harvested for western blot analysis. Cell lysates were separated by SDS-PAGE, transferred to PVDF membranes, and incubated with the indicated antibodies to detect p-TBK1 (Ser172), p-IRF3 (Ser386), ISG60, ISG56, ISG54, RSAD2, EV-A71 VP1, p-STING (Ser366), ISG15 and GAPDH. Anti-myc and anti-Flag antibodies were used to detect cGAS and STING expression. GAPDH was used as a loading control. **b–f**. cGAS-STING-stimulated promoters were suppressed following EV-A71 infection. Eighteen hours post transfection (**a**), *IRF3* (**b**)*, NF-κB* (**c**)*, IL-8* (d)*, IFN-β* (**e**), and *ISRE* (**f**) promoter luciferase activity was measured using a dual-luciferase reporter gene assay. Luciferase induced by cGAS-STING served as a control and was set to 100%. pRL-TK Renilla was used as an internal control. **g** HEK-293T cells were infected with EV-A71 (MOI of 0.5) or mock-infected with virus preservation solution. Twenty-two hours later, cells were transfected with cGAS-STING expression vectors or empty vector. Eighteen hours post-transfection, cells were harvested for total RNA extraction. First strand cDNA synthesis and RT-qPCR analysis with indicated interferon stimulated genes specific primers. Human genes activated by cGAS-STING transfection served as a positive control and were set to 100%. Total RNA was prepared from 293 T cells and analyzed for the transcriptional level of the indicated genes by RT-qPCR. Data in **a**–**g** represent the average of results from three independent experiments (*n* = 3, representative immunoblots are shown). Error bars indicate the standard deviation of the data from three independent experiments. The means and standard deviations are presented. Statistical significance was determined using a two-sided unpaired Student’s *t*-test, *p < 0.05; **p < 0.01; ***p < 0.001

It has been reported that cGAS-STING signaling pathway is frequently suppressed by many viral proteins.^52^ However, whether EV-A71 can inhibit cGAS-STING function is not clear. To address the issue, we activated cGAS-STING in HEK-293T cells using our established system as previously reported.^53^ Exogenous expression of cGAS-STING in HEK-293T cells has also been frequently reported by other research groups to study cGAS-STING triggered innate immune activation.^34,54,55^ Stimulation of the cGAS-STING pathway primed the activation of IRF3 and NF-κB.^53^ Consistent with this idea, IRF3 activation was then observed after cGAS-STING stimulation and was suppressed during EV-A71 infection (Fig. 2a, b). Similarly, NF-κB activation was observed after cGAS-STING stimulation yet was suppressed in the presence of EV-A71 infection (Fig. 2c). As IL-8 is activated by NF-kB,^56^ and we also discovered that the *IL-8* promoter was also activated by cGAS-STING stimulation, but its activation was inhibited in the presence of EV-A71 infection (Fig. 2d). The *IFN-β* promoter contains both IRF3 and NF-κB binding sites and was activated by cGAS-STING stimulation (Fig. 2e). Here, EV-A71 infection inhibited cGAS-STING-stimulated *IFN-β* promoter function (Fig. 2e). Stimulation with cGAS-STING led to IRF3 activation, which was also observed by increased TBK1 and IRF3 phosphorylation (Fig. 2a, Lane 2) when compared with the control (Fig. 2a, Lane 1). EV-A71 infection inhibited cGAS-STING-stimulated TBK1 and IRF3 phosphorylation (Fig. 2a, Lane 4).

Type I interferons are induced during cGAS-STING activation.^34^ Type I interferons induce the production of ISGs.^57^ We further observed that cGAS-STING activation-stimulated ISG production including ISG60, ISG56, ISG54, RSAD2, and ISG15 was inhibited by EV-A71 infection (Fig. 2a), which was further supported by the fact that interferon stimulated response element (ISRE) promoter activity was also activated by cGAS-STING stimulation and suppressed in the presence of EV-A71 infection (Fig. 2f). Various ISG mRNA was also activated by cGAS-STING stimulation and suppressed in the presence of EV-A71 infection (Fig. 2g).

In addition to EV-A71, enteroviruses CV-A16 and EV-D68 infection also induce the production of cGAMP (Supplementary Fig. 3a) and trigger STING activation during viral infection (Supplementary Fig. 3b, c). Furthermore, CV-A16 and EV-D68 infection also markedly inhibited cGAS-STING mediated innate immune activation (Supplementary Fig. 4). Collectively, our data suggest that several enteroviruses have developed the ability to suppress innate immune activation triggered by the cGAS-STING pathway.

### 2A^pro^ EV-A71 inhibits cGAS-STING pathway activation

**Fig. 3:**
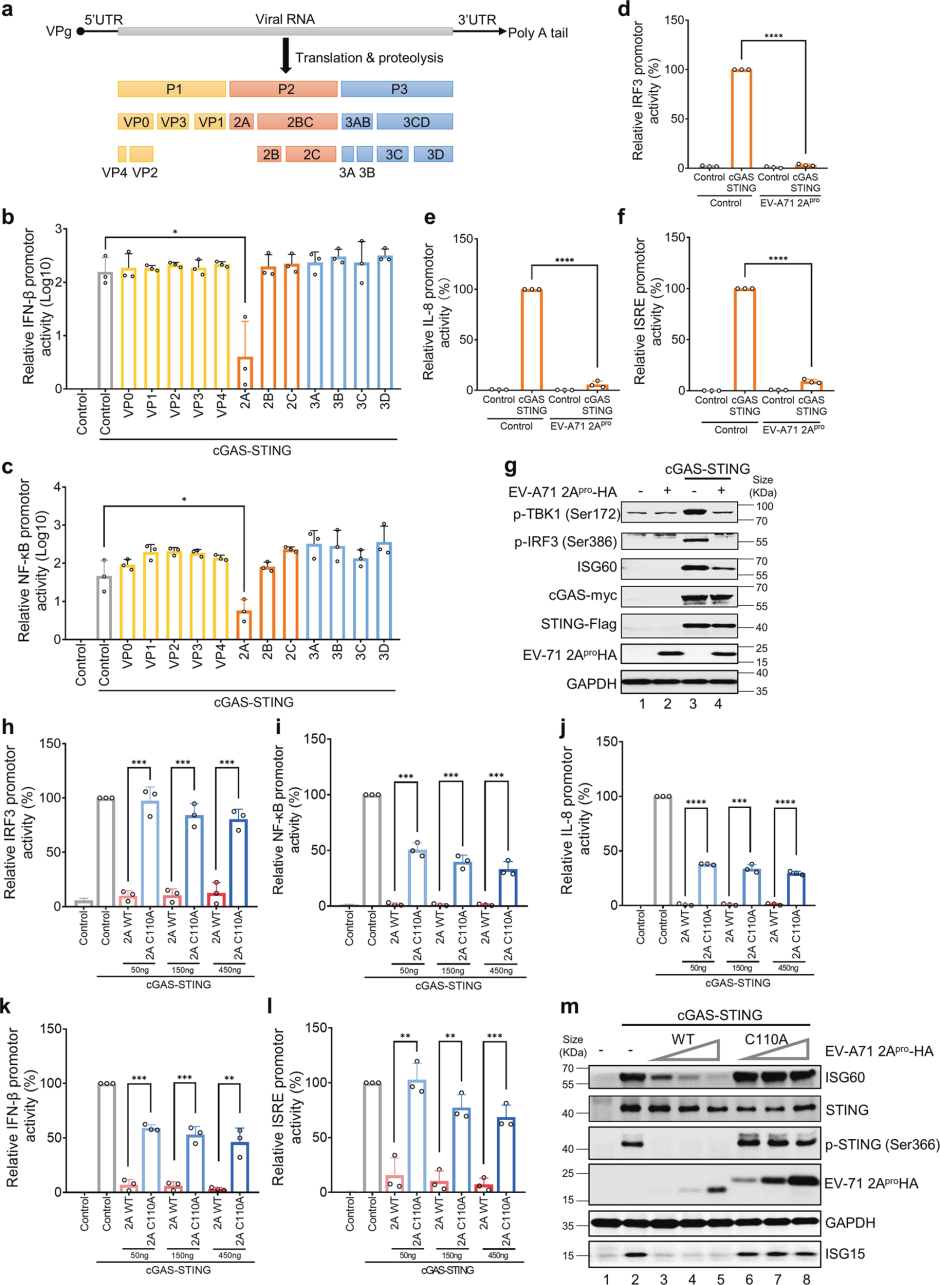
2A^pro^ EV-A71 inhibits cGAS-STING pathway activation. **a** Schematic diagram of the genomic organization and encoded proteins of EV-A71 strain. **b**, **c** EV-A71 2A^pro^ rather than other viral proteins suppressed cGAS-STING-induced *IFN-β* and *NF-κB* promoter activation. HEK-293T cells were co-transfected with *IFN-β* (**b**) or *NF-κB* (**c**) promoter, empty vector, or cGAS-STING expressing vectors, in the presence or absence of EV-A71 VP0, VP1, VP2, VP3, VP4, 2 A, 2B, 2 C, 3 A, 3B, 3 C and 3D expressing vectors. Transactivation of the luciferase reporter gene was determined 18 h after transfection. Luciferase activity induced by cGAS-STING served as a control and was set to 100%. pRL-TK Renilla was used as an internal control. **d**–**f** EV-A71 2A^pro^ inhibited cGAS-STING-induced *IRF3, IL-8* and *ISRE* promoter activation. HEK-293T cells were co-transfected with *IRF3* (D), *IL-8* (E) or *ISRE* (F) promoters, empty vector, or cGAS-STING-expressing vectors, in the presence or absence of EV-A71 2A^pro^ expressing vectors. Eighteen hours post-transfection, cells were harvested for dual-luciferase reporter gene assays. **g** EV-A71 2A^pro^ inhibited cGAS-STING triggered TBK1, IRF3 phosphorylation and ISG60 activation. HEK-293T cells were co-transfected with empty vector, cGAS-STING-expressing vectors, empty vector, or EV-A71 2A^pro^ expressing vector. Eighteen hours post-transfection, cells were harvested for western blot analysis. Anti-myc, anti-Flag, and anti-HA antibodies were used to detect cGAS, STING, and EV-A71 2A^pro^ expression, respectively. p-TBK1 (Ser172), p-IRF3 (Ser386), ISG60, and GAPDH were probed using the indicated antibodies. GAPDH was used as a loading control. **h**–**m** Enzymatic active site of EV-A71 2A^pro^ appears to be important for its inhibitory activity against cGAS-STING function. HEK-293T cells were co-transfected with *IRF3* (**h**), *NF-κB* (**i**), *IL-8* (**j**), *IFN-β* (**k**) or *ISRE* (**l**) promoters, empty vector, or cGAS-STING-expressing vectors, empty vector, or EV-A71 2A^pro^ wild type (WT) or EV-A71 2A^pro^ C110A expressing vectors. Transactivation of the luciferase reporter gene was determined 18 h after transfection. Luciferase activity induced by cGAS-STING served as a control and was set to 100%. pRL-TK Renilla was used as an internal control. **m** HEK-293T cells were co-transfected with empty vector, cGAS-STING-expressing vectors, empty vector, or EV-A71 2A^pro^ WT or C110A expressing vector. Eighteen hours post-transfection, cells were harvested for western blot analysis. Anti-HA antibodies were used to detect EV-A71 2A^pro^ expression. ISG60, STING, p-STING (Ser366), ISG15 and GAPDH were probed using the indicated antibodies. GAPDH was used as a loading control. Data in **b**–**m** represent the average of results from three independent experiments (*n* = 3, representative immunoblots are shown). The error bars indicate the standard deviations of data from three independent experiments. Means and standard deviations are presented. Statistical significance was determined by two-sided unpaired Student’s *t*-test, **p < 0.01; ***p < 0.001; ****p < 0.0001

To identify the viral proteins responsible for EV-A71-mediated cGAS-STING inhibition, we co-expressed cGAS-STING with various EV-A71-encoded proteins in HEK-293T cells (Fig. 3a). We found that 2A^pro^ had the strongest ability to inhibit cGAS-STING-mediated IFN-β and NF-κB promoter activation compared to other EV-A71-encoded proteins (Fig. 3b, c).

Furthermore, cGAS-STING-mediated IRF3 (Fig. 3d) and NF-κB mediated IL-8 (Fig. 3e) activations were remarkably inhibited by 2A^pro^. cGAS-STING activation-stimulated ISRE promoter activity (Fig. 3f) and production of ISG60 was also inhibited by 2A^Pro^ (Fig. 3g). This observation was further supported by the fact that TBK1 and IRF3 phosphorylation were activated by cGAS-STING stimulation and suppressed in the presence of 2A^pro^ (Fig. 3g).

The active enzymatic site of EV-A71 2A^pro^ appears to be important for its inhibitory activity against cGAS-STING function. An EV-A71 2A^pro^ mutant (C110A) had defectiveness to inhibit cGAS-STING-mediated *IRF3* (Fig. 3h), *NF-κB* (Fig. 3i), I*L-8* (Fig. 3j), *IFN-β* (Fig. 3k), and *ISRE* (Fig. 3l) promoter activation. STING phosphorylation, ISG60, and ISG15 induction were effectively suppressed by EV-A71 2A^pro^ but not by EV-A71 2A^pro^ mutant C110A (Fig. 3m).

We observed that EV-D68 2A^pro^ also inhibited STING phosphorylation and ISG60 induction after STING activation (Supplementary Fig. 5a). cGAS-STING-mediated *IRF3* (Supplementary Fig. 5b), *NF-κB* (Supplementary Fig. 5c), *IL-8* (Supplementary Fig. 5d), *IFN-β* (Supplementary Fig. 5e), and *ISRE* (Supplementary Fig. 5f) promoter activations were also inhibited by EV-D68 2A^pro^. Similar results were also obtained for CV-A16 2A^pro^ (Supplementary Fig. 6). Therefore, our observation indicated that 2A^pro^ of several enteroviruses have conserved function of suppressing innate immune activation triggered by the cGAS-STING innate immune pathway.

### TRAF3 is involved in cGAS-STING pathway activation and is a target of 2A^pro^ of EV-A71

**Fig. 4:**
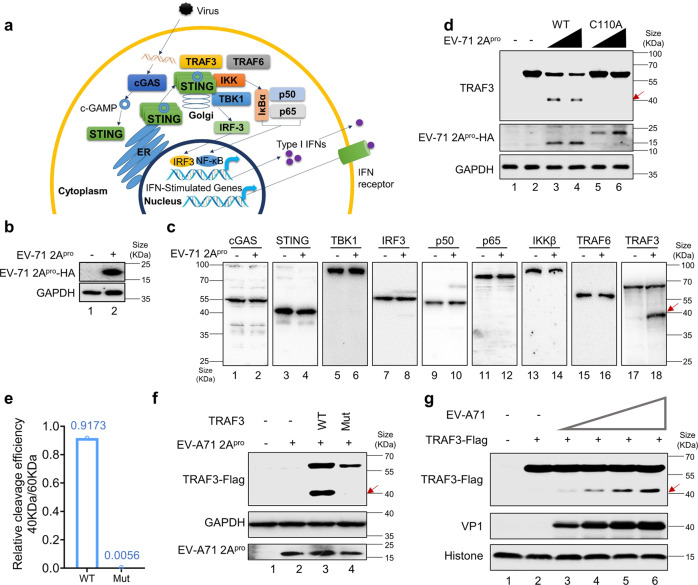
TRAF3 is a target of EV-A71 2A^pro^. **a** Schematic diagram of the process of cGAS-STING activation and factors involved. **b** HEK-293T cells were transfected with empty vector or EV-A71 2A^pro-^expressing vectors. Eighteen hours post-transfection, cells were harvested for western blot analysis. Anti-HA was used to identify EV-A71 2A^pro^. GAPDH was used as a loading control. **c** HEK-293T cells were transfected with empty vector or EV-A71 2A^pro^-expressing vectors (**b**), cGAS, and STING expressing vectors. Eighteen hours post-transfection, cells were harvested for western blot analysis. cGAS, STING, TBK1, IRF3, p50, p65, IKKβ, TRAF6, TRAF3 and GAPDH were detected using the indicated antibodies. GAPDH was used as a loading control. TRAF3 cleavage by EV-A71 2A^pro^ is indicated by an arrowhead. **d** HEK-293T cells were transfected with increasing doses of EV-A71 2A^pro^ WT- or C110A-expressing vectors. Eighteen hours post-transfection, cells were harvested for western blot analysis. Anti-HA and anti-TRAF3 antibodies were used to probe EV-A71 2A^pro^ and TRAF3, respectively. GAPDH was used as a loading control. TRAF3 cleavage by EV-A71 2A^pro^ is indicated by an arrowhead. **e**, **f** TRAF3-WT but not TRAF3-Mut was cleaved by EV-A71 2A^pro^. HEK-293T cells were transfected with empty vector, TRAF3-WT, TRAF3-Mut, and EV-A71 2A^pro^-expressing vectors. Eighteen hours post-transfection, cells were harvested for western blot analysis. Anti-HA was used to identify EV-A71 2A^pro^. Anti-Flag was used to identify TRAF3. GAPDH was used as a loading control. Western blot bands were quantified using ImageJ software and the cleavage efficiencies (defined by the ratio of bind 40KDa/60KDa) of TRAF3-WT and TRAF3-Mut were shown as bar in **e**. **g** HEK-293T cells were transfected with TRAF3-WT and empty vector. Eighteen hours post-transfection, cells were infected with increasing doses of EV-A71. Forty-eight hours post-infection, cells were harvested for western blot analysis. Anti-HA and anti-TRAF3 antibodies were used to probe EV-A71 2A^pro^ and TRAF3. Histone H3 was used as a loading control. TRAF3 cleavage by EV-A71 2A^pro^ is indicated by an arrowhead. Data in **a–g** represent the averages of results from three independent experiments (*n* = 3, representative immunoblots are shown)

We evaluated factors potentially involved in the cGAS-STING pathway as targets of EV-A71 2A^pro^ (Fig. 4a). No EV-A71 2A^pro^-cleaved protein product was detected for cGAS, STING, TBK1, IRF3, IKKβ, p50, p65, or TRAF6 (Fig. 4b, c). In contrast, we detected a clear EV-A71 2A^pro^-cleaved protein product when co-expressed with TRAF3 (Fig. 4c, d). The EV-A71 2A^pro^ mutant (C110A) had an impaired ability to mediate TRAF3 cleavage (Fig. 4d, Lanes 5 and 6) compared to EV-A71 2A^pro^ (Fig. 4d, Lanes 3 and 4). Induced mutations in *TRAF3* reduced the ability of EV-A71 2A^pro^ mediated cleavage of TRAF3 (Fig. 4e, f). TRAF3 was also cleaved during EV-A71 infection (Fig. 4g and Supplementary Fig. 7a). Altogether, these results indicate that TRAF3 is a target of EV-A71 2A^pro^.

**Fig. 5:**
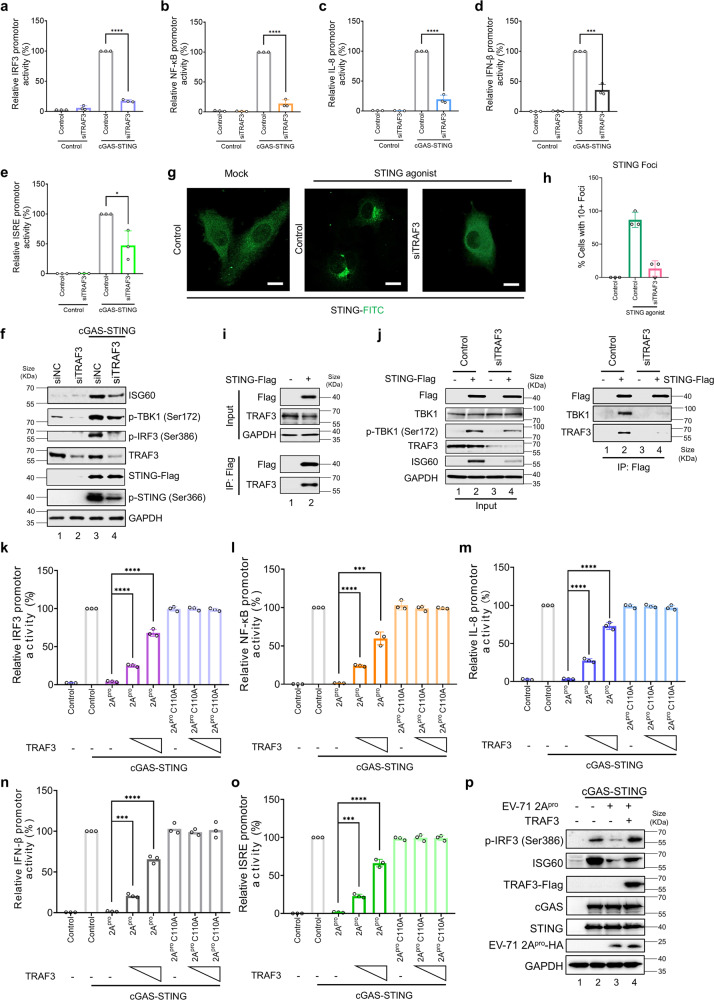
Silencing TRAF3 expression inhibited cGAS-STING activation and TRAF3 rescued cGAS-STING activation from 2A^pro^’s suppression. **a**–**e** HEK-293T cells were transfected with control siRNA (siNC) or si*TRAF3* for 24 h. Next, cells were co-transfected with *IRF3* (**a**), *NF-κB* (**b**), *IL-8* (**c**), *IFNβ* (**d**) or *ISRE* (**e**) promoters, empty vector, or cGAS-STING-expressing vectors. Transactivation of the luciferase reporter gene was determined 18 h after transfection. Luciferase activity induced by cGAS-STING served as a control and was set to 100%. pRL-TK Renilla was used as an internal control. **f** HEK-293T cells were transfected with control siRNA (siNC) or si*TRAF3* for 24 h. Next, cells were co-transfected with empty vector or cGAS-STING-expressing vectors. Eighteen hours post-transfection, cells were harvested for western blot analysis. p-TBK1 (Ser172), p-IRF3 (Ser386), ISG60, TRAF3, p-STING (Ser366), STING and GAPDH were detected using the indicated antibodies. STING was detected by anti-Flag antibody. GAPDH was used as a loading control. **g**, **h** Silencing *TRAF3* decreased the number and intensity of STING foci formation. HeLa cells were transfected with Control siRNA or si*TRAF3*. Twenty-four hours later, control siRNA or si*TRAF3* cells were transfected with STING-Flag. Sixteen hours post-transfection, cells were primed with 100 nM STING agonist or solvent control. Six hours post agonist addition, cells were fixed, treated with Triton-X100, and incubated in 5% FBS, and subcultured to anti-Flag and following FITC Conjugate Goat Anti-Rabbit IgG (H + L) reactivation. Images were captured using a ZEISS laser scanning confocal microscope (Zeiss LSM 900, Oberkochen, Germany). ZEISS ZEN Microscope software was used for acquisition. Percentages of foci formation cells were statistical analyzed. **i** Co-immunoprecipitation analysis of endogenous TRAF3 with STING-Flag. HEK-293T cells were transfected with STING-Flag or a control vector, as indicated. Cell lysates were prepared and immunoprecipitated using anti-Flag beads 36 h after transfection. Precipitated samples were separated by SDS-PAGE, transferred to polyvinylidene fluoride membranes, and reacted with anti-TRAF3 antibodies to detect potential binding of TRAF3 protein and anti-Flag antibodies to detect STING-Flag. GAPDH was used as a loading control. **j** The effect of knocking down TRAF3 on STING-TBK1 interaction. HEK-293T cells were transfected with control siRNA (siNC) or siTRAF3. Twenty-four hours later, HEK-293T cells were co-transfected with cGAS and STING-Flag expression vectors or control vectors for 16 h. Cell lysates were prepared and immunoprecipitated using anti-Flag beads. Precipitated samples were separated by SDS-PAGE, transferred to polyvinylidene fluoride membranes, and reacted with anti-Flag, anti-TBK1, anti-p-TBK1 (Ser172), anti-TRAF3, and anti-ISG60 antibodies to detect indicating proteins. GAPDH was used as a loading control. **k**–**p** HEK-293T cells were transfected with *IRF3* (**k**), *NF-κB* (**l**), *IL-8* (**m**), *IFNβ* (**n**), or *ISRE* (**o**) promoter and cGAS-STING expressing vectors, with empty vector or EV-A71 2A^pro^ WT or EV-A71 2A^pro^ C110A expression vectors, in the presence or absence of vectors expressing TRAF3, as indicated. Transactivation of the luciferase reporter gene was determined 18 h after transfection. Luciferase activity induced by cGAS-STING served as a control and was set to 100%. pRL-TK Renilla was used as an internal control. **p** HEK-293T cells were transfected with cGAS-STING-expressing vectors, empty vector, or EV-A71 2A^pro^ in the presence or absence of vectors expressing TRAF3, as indicated. Eighteen hours post-transfection, cells were harvested for western blot analysis. Cell lysates were separated by SDS-PAGE, transferred to PVDF membranes, and treated with antibodies to detect p-IRF3 (Ser386), ISG60, cGAS, and STING. Anti-Flag antibody was used to detect TRAF3. Anti-HA antibody was used to detect EV-A71 2A^pro^. GAPDH was used as a loading control. Data in **b**–**m** represent the average of results from three independent experiments (*n* = 3, representative immunoblots are shown). The error bars indicate the standard deviations of data from three independent experiments. Means and standard deviations are presented. Statistical significance was determined by two-sided unpaired Student’s *t*-test, **p < 0.01; ***p < 0.001; ****p < 0.0001

To examine the role of TRAF3 in cGAS-STING-induced innate immune activation, we silenced endogenous TRAF3 expression by using small interfering RNA (siRNA). We observed that the knockdown of TRAF3 expression by siRNA significantly reduced the ability of STING activation-induced *IRF3* (Fig. 5a and Supplementary Fig. 7b), *NF-κB* (Fig. 5b and Supplementary Fig. 7c), *IL-8* (Fig. 5c and Supplementary Fig. 7d), *IFN-β* (Fig. 5d and Supplementary Fig. 7e), and *ISRE* (Fig. 5e and Supplementary Fig. 7f) promoter activation. Similarly, STING activation induced STING, IRF3, and TBK1 phosphorylation, as well as the induction of ISG60, was attenuated in the absence of TRAF3 (Fig. 5f and Supplementary Fig. 7g and k). Moreover, we observed that neither the N- nor the C-terminal fragment of TRAF3 cleavage products (Supplementary Fig. 7h) had clear effect on cGAS-STING activation (Supplementary Fig. 7i). Neither the N- nor the C-terminal fragment of TRAF3 cleavage products (Supplementary Fig. 7h) rescued the defect of cGAS-STING activation in TRAF3 depleted cells (Supplementary Fig. 7j).

We also investigated if suppression of TRAF3 would affect STING foci formation. After STING activation by binding to cGAMP, STING translocates from the endoplasmic reticulum (ER) to the ER–Golgi intermediate compartment (ERGIC) and Golgi, and scattered distributed STING transforms to dense foci, which is also termed foci formation.^58^ To address this question, we measured the intracellular distribution of green fluorescent protein (GFP) labeled STING in live cells in response to agonizts. As shown in Fig. 5g, h, STING specific agonizts led to foci formation 6 h later, and TRAF3 silencing decreased the appearance and intensity of STING foci. Furthermore, TRAF3 showed an interaction with STING in HEK-239T cells (Fig. 5i). Depletion of TRAF3 inhibited STING interaction with TBK1 (Fig. 5j), a key regulator of STING phosphorylation and activation.^59^ These data are in keeping with a previous work that STING, TRAF3, and TBK1 form a complex.^60^

EV-A71 2A^pro^ inhibited the cGAS-STING induced *IRF3* (Fig. 5k), NF-*κB* (Fig. 5l), I*L-8* (Fig. 5m), *IFN-β* (Fig. 5n), and *ISRE* (Fig. 5o) promoter activation, which was partially restored by the addition of exogenous TRAF3 (Fig. 5 k–o). Similarly, cGAS-STING-induced IRF3 phosphorylation as well as the induction of ISG60, which was impaired by EV-A71 2A^pro^, could be partially rescued by exogenous TRAF3 (Fig. 5p). Together, these results suggested that TRAF3 is an important factor in the cGAS-STING activation pathway and an important target of EV-A71 2A^pro^.

### 2A^pro^ of EV-A71 inhibits the anti-viral function of cGAS-STING activation

**Fig. 6:**
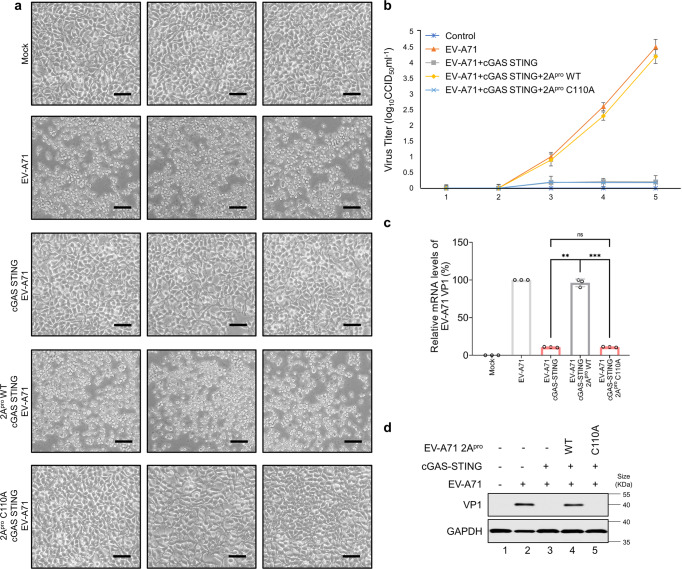
**2**A^pro^ of EV-A71 inhibits the anti-viral function of cGAS-STING activation. **a** CPE induced by EV-A71. HEK-293T cells were transfected with empty or cGAS-STING-expressing vectors in the presence or absence of exogenous EV-A71 2A^pro^ WT or C110A. Twelve hours later, the cells were infected with equal amounts (MOI of 0.5) of EV-A71 for 6 h. The cells were then washed and incubated with fresh culture medium. Five days later, the cells were observed for morphological changes and photographed using light microscopy at 400 × magnification. Scale bar represents 50 μm. **b** EV-A71 from HEK-293T cells was collected on days 1–5 after infection (A). The viral titer was determined using CCID50 assays. **c** The viral RNA levels of EV-A71 were evaluated by RT-qPCR using SYBR green. Primers targeted EV-A71 VP1 to monitor viral replication. GAPDH abundance was used as a control. **d** EV-A71 from HEK-293T cells was collected on day 5 after infection for western blot. Cell lysates were separated by SDS-PAGE, transferred to PVDF membranes, and treated with the indicated antibodies to detect EV-A71 VP1 and GAPDH. GAPDH was used as a loading control. Data in **a**–**c** represents the average of results from three independent experiments (*n* = 3, representative immunoblots are shown). Error bars indicate the standard deviation of the data from three independent experiments. Means and standard deviations are presented. Statistical significance was determined using two-sided unpaired *t*-test, *p < 0.05; **p < 0.01; ***p < 0.001; ****p < 0.0001

We evaluated whether STING pathway activation antagonizes EV-A71 infection. HEK-293T cells were co-transfected with STING- and cGAS-expressing plasmids to mimic STING and downstream gene activation. At 18 h post-transfection, cells were then infected with EV-A71 for 12 h, washed, and defined as day 0 (Fig. 6a). Both EV-A71 viral titers (Fig. 6b) and viral VP1 levels (Fig. 6c, d) were suppressed in the presence of STING activation. Cells transfected with empty vector exhibited a significant cytopathic effect (CPE) 5 days post-infection (Fig. 6a). In contrast, STING-activated cells showed no apparent CPE (Fig. 6a). Moreover, in TRAF3 depleted cells, the anti-viral function of cGAS-STING was attenuated (Supplementary Fig. 8). When 2A-resistant TRAF3 was expressed in TRAF3 depleted HEK-293T cells, the anti-viral function of cGAS-STING was restored (Supplementary fig. 8). Our data reinforced the crucial role of TRAF3 in cGAS-STING activation and indicated that the importance of STING activation in combatting EV-A71 infections in humans.

We have demonstrated above that EV-A71 2A^pro^ could suppress STING activation-triggered innate immune responses. Next, we tested whether EV-A71 2A^pro^ could effectively inhibit STING activation-induced antiviral activity against EV-A71. Over the next 5 days, supernatants were harvested each day for titer analysis, and cells were observed under a microscope. As shown in Fig. 6, compared to STING activation-suppressed EV-A71 replication, 2A^pro^ remarkably rescued cells from EV-A71-induced CPE (Fig. 6) and restored EV-A71 viral replication (Fig. 6). However, EV-A71 2A^pro^ mutant (C110A) could not neutralize the anti-viral activity mediated by cGAS-STING activation (Fig. 6). Thus, inhibiting EV-A71 2A^pro^ function represents a promising anti-viral strategy.

## Discussion

EV-A71 is a non-enveloped virus bearing a capsid with icosahedral symmetry containing a non-segmented positive-sense single-stranded genomic RNA. EV-A71 viral infection could trigger RLR^61,62^ and TLR^63,64^ sensing owing to viral genomic RNA detection by the host. The mechanism of viral evasion has been well investigated.^26,31,32,65,66^ Another important pathogen pattern recognition pathway mediated by cGAS-STING recognizes intracellular DNA fragments, thus representing host defenses against DNA viruses.^67^ As a non-enveloped RNA virus, it is not known whether EV-A71 infection triggers cGAS-STING activation. Results from this work indicated that EV-A71 infection can trigger innate immune responses through STING activation. We observed that EV-A71 infection-triggered STING activation is cGAS dependent (Supplementary Fig. 1). Knocking out cellular cGAS expression prevented EV-A71 triggered phosphorylation of STING and downstream process of innate immune activation (Supplementary Fig. 2). Inactivated EV-A71 failed to prime cGAS-STING activation (Supplementary Fig. 1). According to our current data, EV-A71 infection induced mitochondrial damage. Analysis of the mitochondrial membrane potential using JC-1 staining uncovered that EV-A71 infection decreased the intensity of red fluorescence (590 nm) and increased the intensity of green fluorescence (529 nm) in THP-1 cells, indicating mitochondrial damage (Fig. 1j). Consequently, we detected the discharge of mtDNA into the infected cells’ cytoplasm and the production of 2'3'-cGAMP, an indication of the activation of cGAS (Supplementary Fig. 1). Suppression of mtDNA release or mtDNA exhaustion prevented EV-A71 infection triggered activation of STING (Supplementary Fig. 1).

Several research groups have reported that EV-A71 infection could not trigger RLR-mediated immune responses in HEK-293T,^31^ HeLa,^25,32,33^ RD^31^ cells. Interestingly, naked EV-A71 RNA triggered RLR pathway activation in HeLa cells,^25^ suggesting that EV-A71 particles have mechanisms for RLR evasion.^31,32^ We have confirmed that EV-A71 infection did not trigger RLR-mediated immune activation in HEK-293T cells (Fig. 1k) or STING silenced THP-1 cells (Fig. 1e). However, when STING and cGAS molecules were expressed in HEK-293T cells, these cells showed STING-mediated immune activation (STING phosphorylation, IRF3 phosphorylation, and ISG induction) after EV-A71 infection (Fig. 1k). These gain-of-function experiments indicate that EV-A71 infection could avoid RLR pathway activation and remain sensitive to cGAS-STING-mediated innate immune activation.

We have also demonstrated that MAVS which is critical for RLR pathway activation was dispensable for EV-A71 infection-triggered STING phosphorylation (Supplementary fig. 1g). At the same time, depletion of TRAF3 inhibited EV-A71 infection triggered STING phosphorylation (Supplementary Fig. 7k). These results reinforce the important and specific role of TRAF3 in STING activation observed in our experimental system.

In addition to EV-A71, other enteroviruses EV-D68 and CV-A16 infection also triggered STING activation (Supplementary Fig. 3). Suppression of cGAS-STING activation during these enteroviruses infection was observed (Supplementary Fig. 4). To our knowledge, this is the first example of a non-enveloped RNA virus that can trigger STING activation. Previously, enveloped RNA viruses, such as influenza^43,68^ and coronaviruses,^48^ have been shown to trigger cGAS dependent STING activation after viral membrane and host cell membrane fusion.^37,38^ Also, cGAS independent mechanism of STING activation have also been reported.^43^ Hemagglutinin fusion peptide of influenza A virus interacts with STING and primed STING activation.^43^ SARS-CoV-2 has also been reported to trigger mitochondrial damage and mtDNA release during viral infection, which subsequently prime cGAS-STING activation.^42^ Other studies proposes that cell fusion damaged nuclei and resulted in the formation of micronuclei that were sensed by cGAS and led to the activation of STING.^38^ Different viruses have developed different strategies for suppressing STING activation.^43,47,48,68^

We also discovered that EV-A71 has evolved a strategy to limit STING activation. Among the 12 EV-A71 encoded proteins, 2A^pro^ strongly inhibited STING activation (Fig. 3). Addition of 2A^pro^ prior to EV-A71 infection inhibited STING mediated suppression of EV-A71 replication (Fig. 6). It is known that 2A^pro^ antagonizes the RLR pathway by inducing the cleavage of MAVS^32^ and decreases the expression of the RNA sensor MDA5.^26^ EV-A71 2A^pro^ also inhibits TLR signaling by reducing the expression of TLR3.^15^ STING activation triggers the phosphorylation of TBK1, STING, and IRF3.^59^ Phosphorylated IRF3 molecules translocate to the nucleus to boost type I interferons and induce the production of ISGs. EV-A71 2A^pro^ inhibited STING activation-triggered phosphorylation of TBK1, STING, and IRF3 (Fig. 3g). Consequently, STING activation-triggered ISG induction is inhibited by EV-A71 2A^pro^. The viral protease 2A^pro^ from EV-D68 and CV-A16 also had the ability to inhibit STING activation (Supplementary Fig. 5 and 6), suggesting an evolutionarily conserved function of these viral proteins. As mutant of enzymatic center of EV-A71 2A^pro^ showed defective suppression of STING activation when compared to the wild type 2A^pro^, the enzymatic activity of 2A^pro^ is essential for STING-mediated immune function inhibition (Fig. 3h–m).

Evaluation of host factors potentially involved in STING activation identified TRAF3 as a target of 2A^pro^ (Fig. 4). Important roles for TRAF3 in TLR3-, TLR4-, and TLR9- triggered type I IFN production has been reported.^69,70^ TRAF3 has also been shown to be involved in RLR-mediated immune activation.^71,72^ TRAF3 is an important RIG-I co-factor for the induction of IRF3 phosphorylation and downstream type I IFN production.^73,74^ We discovered that TRAF3 is also important for STING activation. TRAF3 showed interaction with STING in co-immunoprecipitation (Fig. 5i). Silencing TRAF3 inhibited STING-TBK1 interaction, TBK1 and IRF3 phosphorylation and downstream signaling (Fig. 5). Consequently, *ISGs* transcription was suppressed (Fig. 5). Addition of exogenous TRAF3 rescued STING activation from 2A^pro^ mediated suppression (Fig. 5). STING activation is triggered by binding to 2'3'-cGAMP and the subsequent formation of foci in the ERGIC and Golgi.^58^ We observed that TRAF3 was important for STING foci formation (Fig. 5). EV-A71 2A^pro^ suppresses STING activation by inducing TRAF3 cleavage (Fig. 4). Cleaved TRAF3 fragments showed no function of supporting STING activation (Supplementary Fig. 7). Knocking down TRAF3 impaired STING activation mediated anti-EV-A71 replication. Addition of 2A^pro^ cleavage resistant TRAF3 mutant proteins rescued STING triggered anti-viral effect from 2A^pro^ mediated suppression (Supplementary Fig. 8). Activation of cGAS-STING pathway has been shown to mediate broad viral suppression against bacteria, DNA viruses, SARS-CoV-2, and retroviruses. EV-A71 2A^pro^ mutant C110A could not neutralize the anti-viral function of cGAS-STING activation (Fig. 6). Since STING activation prior to EV-A71 infection could generate strong cellular immunity against EV-A71 infection, restoring STING function by inhibiting EV-A71 2A^pro^-mediated TRAF3 cleavage could be a promising new anti-viral therapeutic drug target.

## Materials and methods

### Cell lines and viruses

The epithelial kidney cells Vero (Cat No. CCL-81, ATCC), human embryonic kidney cell line HEK-293T (Cat No. CRL-3216, ATCC, Virginia, USA), HEK-293T-STING stable cell line, were maintained in Dulbecco’s modified Eagle’s medium (Cat No. BC-M-005, Bio-Channel, Nanjing, China) supplemented with 10% fetal bovine serum (Cat No. BC-SE-FBS01, Bio-channel). HEK-293T-STING cells were cultured under puromycin selection. The human cervical carcinoma cell line HeLa (Cat No. CRM-CCL-2, ATCC) was maintained in Eagle’s Minimum Essential medium (Cat No. BC-M-005, Bio-Channel, Jiangsu, China) supplemented with 10% fetal bovine serum (Cat No. BC-SE-FBS01, Bio-Channel, Jiangsu, China). THP-1 human leukemic monocyte cells (Cat No. TIB-202, ATCC), NCM460 normal human colon mucosal epithelial cells (Cat No. HTX1841, ATCC), THP-1 and THP-1 cGAS^KO^ cells were maintained in RPIM-1640 medium (Cat No. BC-M-017, Bio-Channel, Jiangsu, China) supplemented with 10% fetal bovine serum. HUVEC human umbilical vein endothelial cells (Cat No.CRL-1730, ATCC) were maintained in complete Endothelial Cell medium (ScienCell^TM^, Carlsbad, CA). Human primary colonic mucosal epithelial cells (purchased from Meisen Chinese Tissue Culture Collections, Zhejiang, China) were maintained in complete Primary epithelial cell medium (CTCC-001-PriMed, Meisen, Hangzhou, China). THP-1-cGAS^KO^ cells were kindly provided by Guofei (Chinese Academy of Medical Sciences, Beijing, China). The cell lines were cultured in a humidified atmosphere in 5% CO_2_ at 37 °C. Phosphate-buffered saline (PBS) used in cell culturing was purchased from Bio-Channel (Cat No. BC-BPBS-01, Bio-Channel, Jiangsu, China).

The Changchun (CC) strains of EV-A71 viruses (CC063) were isolated from throat swabs of patients with HFMD in Changchun, China, in 2010.^75,76^ EV-D68 circulating strains from the 2014 United States outbreak, US/KY/14-18953 (ATCC, VR-1825D), were described previously.^77^ The CC024 strains of CV-A16 were isolated from the throat swabs of patients with HFMD in 2010 and described previously.^78^ Viruses were continuously subcultured in Vero until the 10th passage to ensure stable titers and genetic features. When the CPE cells reached 90%, viruses were collected through centrifugation and filtration. Viral titers were determined in Vero cells using the microplate CPE method and calculated using the Reed–Muench method.^75^

### Antibodies and reagents

Anti-TRAF3 (rabbit / IgG, Cat No. 18099-1-AP), anti-STING (rabbit / IgG, Cat No. 19851-1-AP), anti-cGAS (rabbit / IgG, Cat No. 26416-1-AP), anti-ISG60 (rabbit / IgG, Cat No. 15201-1-AP), anti-MYC-tag (rabbit / IgG, Cat No. 16286-1-AP), anti-GAPDH (rabbit / IgG, Cat No. 10494-1-AP), anti-ISG15 (rabbit / IgG, Cat No.15981-1-AP), anti-MDA5 (rabbit / IgG, Cat No.21775-1-AP), anti-DDX58 (rabbit / IgG, Cat No.20566-1-AP), anti-ISG56 (rabbit / IgG, Cat No.23247-1-AP), anti-MAVS (rabbit / IgG, Cat No.14341-1-AP), anti-ISG54 (rabbit / IgG, Cat No.12604-1-AP), anti-RSAD2 (rabbit / IgG, Cat No.11833-1-AP), anti-Histone H3 (rabbit / IgG, Cat No.17168-1-AP) antibodies were purchased from Proteintech (Illinois, USA). Anti-Phospho-TBK1/NAK (Ser172) (rabbit / IgG, Cat No. #5483), anti-Phospho-IRF-3 (Ser396) (rabbit mAb, Cat No. #4947), anti-TBK1/NAK (rabbit mAb, Cat No. #38066), and anti-IRF-3 (rabbit mAb, Cat No. #11904), anti-Phospho-STING (rabbit / IgG, Cat No.50907 s) antibodies were acquired from Cell Signaling Technology (Massachusetts, USA). Mouse monoclonal anti-FLAG monoclonal M2 antibody (Cat No. F1804) was obtained from Sigma-Aldrich (St. Louis, Missouri, USA). The mouse monoclonal anti-HA (hemagglutinin) antibody (Cat No. 901514) was purchased from BioLegend (California, USA). Anti-VP1 (rabbit / IgG, Cat 43908) was purchased from GeneTex (California, USA). STING agonist (Cat No. HY-103665, CAS No.: 2138299-29-1) and DDC (Cat No. HY-17392, CAS No.: 7481-89-2) were purchased from MedChemExpress (Shanghai, China). VBIT-4 (Cat No. T13287, CAS No.: 2086257-77-2) was purchased from TOPSCIENCE (Shanghai, China).

### Plasmids

The pEF-BOS STING-Flag-His, cGAS-myc-VR1012, *NF-κB* promoter, *IFN-β* promoter, *IL-8* promoter, *IRF3* promoter and *ISRE* promoter luciferase reporter plasmids were described previously.^47^ All EV-A71 protein constructs utilized in this study have been described previously,^79^ including VP0, VP1, VP2, VP3, VP4, 2 A,2 A C110A, 2B, 2 C, 3 A, 3B, 3 C, and 3D. Flag tagged TRAF3, TRAF3 mutant G435A & K436A (2A^pro^ natural recognition site during EV-A71 virus assembly and replication) and TRAF3 cleavage products 1-435, 436-568 were cloned into the VR1012 vector. All plasmids were sequenced with Sanger sequencing and the correct insertion of each gene sequence was verified. The DNA plasmids were transfected into HEK-293T cells using Hieff Trans™ Liposomal Transfection Reagent (Yeasen Biotech, Shanghai, China) according to the manufacturer’s instructions.

### Cell transfection

Empty plasmids or various plasmids were transfected with the Hieff Trans™ Liposomal Transfection Reagent (Cat No. 40802ES08, Yeasen Biotech, Shanghai, China) according to the manufacturer’s instructions. HEK-293T STING stable expression cell lines were constructed using the Lenti-X™ Expression System (Cat No. 632164, Takara, Osaka, Japan). THP-1 Control/shSTING shut-down cell lines were constructed using the Lenti-X™ shRNA Expression Systems (Cat No. 632177, Takara, Osaka, Japan). TRAF3 scrambled siRNAs were synthesized by RiboBio (Guangzhou, China). siRNA was transfected using Lipofectamine RNAiMAX (Invitrogen, California, USA) according to the manufacturer’s protocols.

### CPEs observation

To observe CPEs, HEK-293T cells were seeded in a cell culture dish and infected with EV-A71 of indicated multiplicity of infection (MOI). Morphological changes were observed and photographed using a light microscope at ×400 magnification.

### Viral titer determination

Virus titers were characterized using the median endpoint of the cell culture infectious dose (CCID50). Serially diluted viruses containing supernatants were added to Vero cells cultured in 96-well plates and eight replicates were used for each dilution. CCID50 values were determined by counting infected Vero cell culture wells with obvious CPEs and calculated using the Reed–Muench method.

### Western blotting

Cells were collected, washed twice with pre-cooled PBS, and lysed in lysis buffer (Cat No. P0013, Beyotime, Shanghai, China) supplemented with complete protease inhibitor cocktail tablets (Roche, Basel, Switzerland) at 4 °C for 20 min, and then mixed with 5 × loading buffer (Cat No. FD002, Fude, Zhejiang, China) and boiled for 30 min. Cell lysates were centrifuged at 13,000 rpm at 4 °C for 5 min. Supernatant were loaded and separated by sodium dodecyl sulfate-polyacrylamide gel electrophoresis (SDS-PAGE). Separated proteins on gel were transferred to PVDF membranes (IPVH00010, Millipore, Ireland) using a wet apparatus (Bio-Rad, California, USA).^80^ The membranes were incubated overnight with primary antibody dilutions. Next day, membranes were washed with TBST for three times and then incubated in secondary antibody dilutions (1:5000) for 1 h (Cat No. HA1006 or HA1001, HuaBio, Hangzhou, China). Exposure was performed with SignalBright Pro Chemiluminescent Substrate (Cat No. PK10011, Proteintech, Illinois, USA) following the manufacturer’s protocol.

### Real-time quantitative PCR (RT-qPCR)

Total RNA was obtained using the TRIzol Reagent (Life Technologies, California, USA) following the manufacturer’s instructions, and the extracted RNA served as templates of first strand cDNA synthesis using M-MLV reverse transcriptase (Takara, Osaka, Japan). The generated cDNA was used as a template to detect EV-A71 RNA and host cellular mRNA expression. SYBR Premix Ex Taq reagent (Takara, Osaka, Japan) was used to quantify RNA copy numbers. The glyceraldehyde-3-phosphate dehydrogenase (*GAPDH*) gene was used as an internal control. The LightCycler480 (Roche) were used for real-time quantification PCR amplification. Amplification reactions conditions: 95 °C for 30 s, 40 cycles of 95 °C for 5 s, and 60 °C for 34 s, and dissociation. Single peaks identified in the melting curve analysis indicated the presence of a specific amplicons. Specific primers were used to amplify target genes cDNA. Relative mRNA expression was calculated with the comparative cycle threshold (2 − ΔΔCt) method. Experiments were independently repeated three times. The qPCR primers used in our experiments were listed as follows: *EV-A71 VP1*-F: 5'-ATGATGGGHACNTTCTC-3', *EV-A71 VP1*-R: 5'-GANTTNCCDGCRTAVTTTGG-3', *IFIT2*-F: 5'-AAGCACCTCAAAGGGCAAAAC-3', *IFIT2*-R: 5'-TCGGCCCATGTGATAGTAGAC-3', *IFIT3*-F: 5'-TCAGAAGTCTAGTCACTTGGGG-3', *IFIT3*-R: 5'-ACACCTTCGCCCTTTCATTTC-3', *IFIT1*-F: 5'-TTGATGACGATGAAATGCCTGA-3', *IFIT1*-R: 5'-CAGGTCACCAGACTCCTCAC-3', *IFIH1*-F: 5'-TCGAATGGGTATTCCACAGACG-3', *IFIH1*-R: 5'-GTGGCGACTGTCCTCTGAA-3', *OASL*-F: 5'-CTGATGCAGGAACTGTATAGCAC-3', *OASL*-R: 5'-CACAGCGTCTAGCACCTCTT-3', *DDX58*-F: 5'-CTGGACCCTACCTACATCCTG-3', *DDX58*-R: 5'-GGCATCCAAAAAGCCACGG-3', *HERC5*-F: 5'-GGTGAGCTTTTTGCCTGGG-3', *HERC5*-R: 5'-TTCTCCGGCAGAAATCTGAGC-3', *D-loop*-MT325F: 5'-CACAGCACTTAAACACATCTCTGC-3', *D-loop*-MT474R: 5'-AGTATGGGAGTGRGAGGGRAAAA-3'.

### Luciferase activity

HEK-293T cells (1 × 10^5^) were plated in 24-well dishes 24 h before transfection. One hundred and fifty nanograms reporter gene plasmids for *IFN-β, NF-κB, IRF3, IL-8*, or *ISRE*-Luc promoter, 7 ng Renilla luciferase plasmid (pRL-TK), and other expression vectors were transfected with Hieff Trans™ Liposomal Transfection Reagent (Yeasen Biotech). Sixteen hours post transfection, cells were collected and lysed with the Passive Lysis Buffer (Cat No. E1941; Promega, Wisconsin, USA). Firefly or Renilla luciferase activities were detected using a Dual-Glo Luciferase assay system (Cat No. E2920; Promega, Wisconsin, USA). Firefly luciferase activities represent primary reporter expression and Renilla luciferase activity was used for reporter normalization.

### Confocal microscopy

Transfected HeLa cells were sub cultured and grown on coverslips. To detect STING status and cellular location, cells were fixed with 4% paraformaldehyde, permeated with Triton-X100, and incubated in 5% FBS, and incubated in anti-Flag and following FITC Conjugate Goat Anti-Rabbit IgG (H + L) reactivation at room temperature. Cell images were captured by ZEISS laser scanning confocal microscope (Zeiss LSM 900, Oberkochen, Germany). The ZEISS ZEN Microscope software was used for operation and acquisition. Percentages of foci formation cells were statistical analyzed.

### Mitochondrial activity

Mitochondrial activity was defined by membrane potential which was visualized using the JC-1 staining (JC-1 MitoMP Detection Kit Dojindo, MT09, Kumamoto, Japan). Images were captured using a ZEISS laser scanning confocal microscope (Zeiss LSM 900). A ZEISS ZEN Microscope software was used for operation.

### Isolation of mitochondria and measurement of ratio of *D-loop*

EV-A71 infected or mock-infected cells were collected by 500 × *g* centrifugation for 5 min at 4 °C, cell pellets were washed once with pre-cooled PBS and resuspended in cold hypotonic buffer with protease inhibitors (complete protease inhibitor cocktail tablets (Roche)) added and were then lysed by Dounce homogenization.^81^ Cell lysates were centrifuged at 500 × *g* for 10 min at 4 °C to remove nuclear pellets, and the supernatants were transferred to a new Eppendorf tube and then centrifuged at 5000 × *g* for 10 min at 4 °C to obtain the mitochondrial fraction (P5K) and the nonmitochondrial cytosolic fraction (S5K). mtDNA was purified from different fractions with DNA extraction Kit (D3396-01, OMEGA, Norcross, Georgia) and followed to real-time qPCR analysis using specific *D-loop* primers.^82^ The ratio of S5K to P5K represent the relative discharge of mtDNA to the cytosol from mitochondria.

### Detection of 2'3'-cGAMP content in cells

EV-A71-infected cells were harvest with pre-cooled PBS and lysed in M-PER™ lysis buffer (78501, Thermo Fisher). 2'3'-cGAMP was measured with a competitive ELISA assay (501700, Cayman Chemical). This assay is based on the competition between native 2'3'-cGAMP in samples and a defined dose of 2'3'-cGAMP-horseradish peroxidase (HRP) conjugate (2'3'-cGAMP-HRP Tracer) for a limited amount of 2'3'-cGAMP Polyclonal Antiserum. The quantification was operated according to the manufacturer instructions.

### Immunoprecipitation (Co-IP)

HEK-293T cells were seeded onto 10-cm dishes and transfected with empty plasmids, expression plasmids, siRNA using the Hieff Trans™ Liposomal Transfection Reagent (Yeasen Biotech) or Lipofectamine RNAiMAX (Invitrogen, California, USA) complying with the manufacturer’s instructions. At 24 h after transfection, the cells were harvest with pre-cooled PBS and lysed in lysis buffer (150 mM NaCl, 0.5% NP-40, and complete protease inhibitor cocktail tablets (Roche) with 50 mM Tris (pH 7.5)) on shakers at 4 °C for 60 min. Cell lysates were then centrifuged at 10,000 × *g* at 4 °C for 30 min. Supernatant were incubated with anti-Flag affinity matrix (A-2220; Sigma-Aldrich) at 4 °C for 12 h on a rotator. Matrix was then washed six times with wash buffer (20 mM Tris (pH 7.5), 100 mM NaCl, 0.05% Tween-20 and 0.1 mM EDTA). Following 6th wash, 50 μl elution buffer (100 mM glycine-HCl (pH 2.5)) was used to resuspend the matrix, incubated for 5 min at room temperature. The matrix was then centrifuged for 1 min at 6000 rpm and supernatant containing the eluted proteins were obtained.

### Statistical analysis

Data presented used mean values ± standard error (mean ± SE) of three independent experiments. Two-tailed Student’s *t*-tests were introduced to analyze the significance of the potential differences. Differences were considered to be statistically significant at *P < 0.05, and highly significant at **P < 0.01, ***P < 0.001 and ****P < 0.0001.

## Supplementary information

Supplementary Figure

Original films of Western blot

## Data Availability

Data generated and analyzed in the current study are included in this manuscript and supplementary files.
