# Supplementary material for: TRAF3 activates STING-mediated suppression of EV-A71 and target of viral evasion
Source: Signal Transduct Target Ther. 2023 Feb 24;8:79. doi: 10.1038/s41392-022-01287-2 (PMC9950063; doi:10.1038/s41392-022-01287-2)
Supplement: Supplementary file 1 — Supplementary Figure [file 41392_2022_1287_MOESM1_ESM.docx]

Supplementary Materials for

TRAF3 as an activator of STING mediated suppression of EV-A71 and target of viral evasion

Wenwen Zheng^1^, Zhenbang Zhou^1^, Yajuan Rui^1^, Runxin Ye^1^, Fengyan Xia^1^, Fei Guo^3^, Xiaoman Liu^3^, Jiaming Su^1^, Meng Lou^1^, and Xiao-Fang Yu*^1,2^

1 Cancer Institute (Key Laboratory of Cancer Prevention and Intervention, China National Ministry of Education), The Second Affiliated Hospital, Zhejiang University School of Medicine, Hangzhou, Zhejiang 310009, China;

2 Cancer Center, Zhejiang University, Hangzhou, Zhejiang 310058 China

3 National Health Commission of the People’s Republic of China Key Laboratory of Systems Biology of Pathogens, Institute of Pathogen Biology and Center for AIDS Research, Chinese Academy of Medical Sciences and Peking Union Medical College, Beijing 100730, China.

Correspondence: Xiao-Fang Yu (E-mail: [xfyu1@zju.edu.cn](mailto:xfyu1@zju.edu.cn))

These authors contributed equally: Wenwen Zheng, Zhenbang Zhou, Yajuan Rui and Runxin Ye

**This PDF file includes:**

Figures. S1 to S8


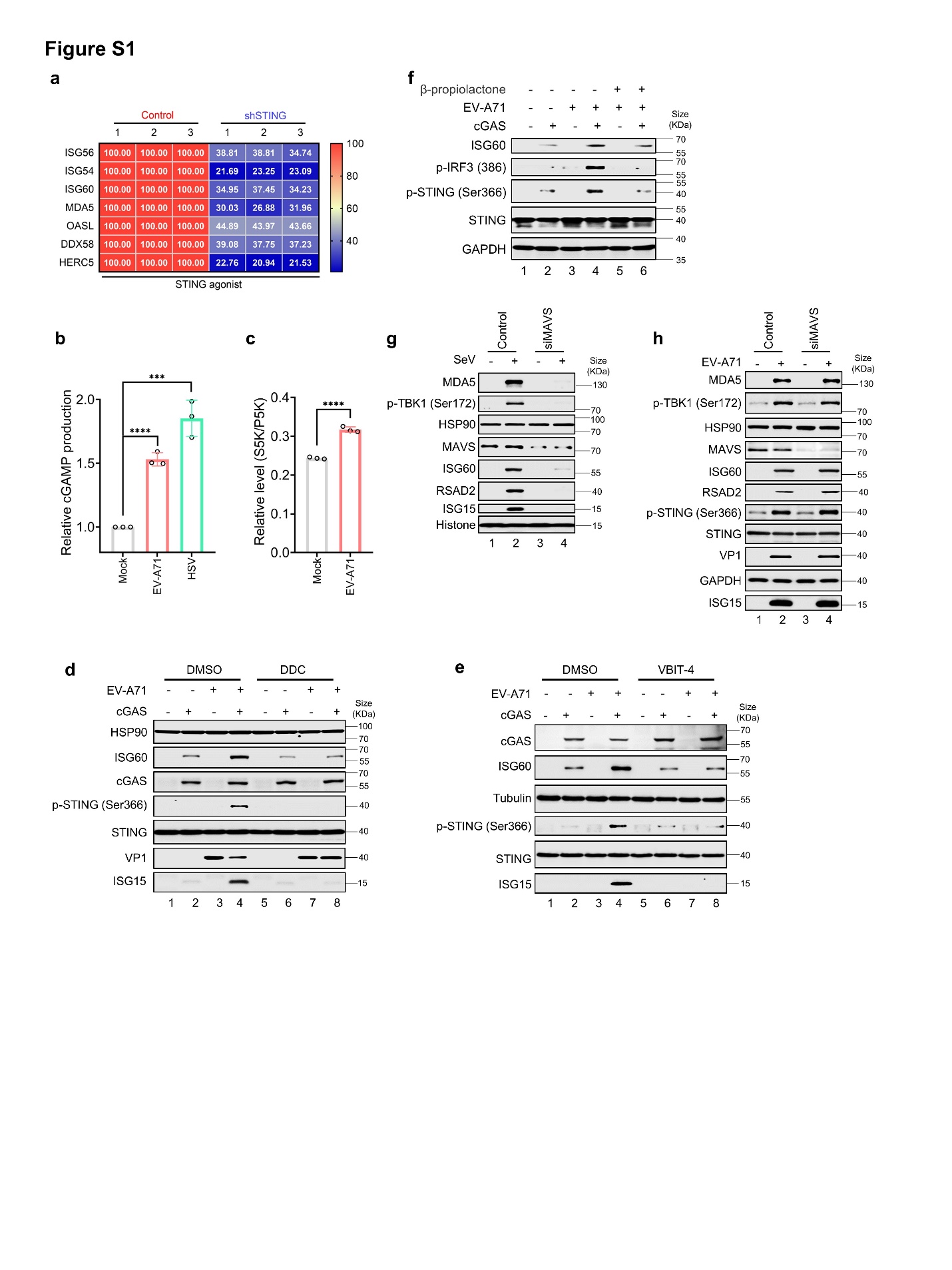


**a.** Inhibition of STING agonist-stimulated human genes by silencing STING expression. THP-1 control or shSTING cells were treated with 100 nM STING agonist for 5 h and harvested for total RNA extraction, first strand cDNA synthesis, and RT-qPCR analysis with indicated interferon stimulated genes specific primers. Human genes activated by STING agonist alone served as a positive control and were set to 100%. Total RNA was prepared from THP-1 cells and analyzed for the transcriptional level of the indicated genes by RT-qPCR (n=3 independent biological experiments). Statistical significance was determined by two-sided unpaired t-test. **b.** 2’3’-cGAMP levels in THP-1 cells infected with EV-A71 at a multiplicity of infection (MOI) of 0.5. THP-1 cells were infected with EV-A71 for 2 h and harvest for further analysis. A competitive ELISA assay determined the 2’3’-cGAMP concentrations. **c.** THP-1 cells were infected with EV-A71 at an MOI of 0.5 for 2h. Cell lysates were collected and then separated to S5K, nonmitochondrial cytosolic fraction, P5K, mitochondrial fraction, by differential centrifugation. Mitochondrial DNA extracted from each cell fractions were evaluated by Quantitative Real-time PCR. The data are expressed as ratio of D-loop DNA levels between S5K and P5K. **d.** HEK-293T STING stable cell line were transfected with cGAS expression vectors or empty vectors. After 36h, transfected cells were treated with DDC (20µmol/mL) or constant volume DMSO for 48h. Cells were then mock-infected or infected with EV-A71 at an MOI of 0.5 for 4h and harvested for western blot analysis. cGAS, ISG60, p-STING (Ser366), STING, ISG15 were probed with indicated antibodies, and HSP90 was used as a loading control. **e.** HEK-293T STING stable cell line were transfected with cGAS expression vectors or empty vectors. After 36h, transfected cells were treated with VBIT-4 (1 µmol/mL) or constant volume DMSO for 24h. Cells were then mock-infected or infected with EV-A71 at an MOI of 0.5 for 4h and harvested for western blot analysis. cGAS, p-STING(Ser366), and STING were probed with indicated antibodies, Tubulin was used as a loading control. **f.** HEK-293T STING stable cell line were transfected with cGAS expression vectors or empty vectors. After 36 h, those transfected cells were then mock-infected or infected with EV-A71 at an MOI of 0.5 or β-propiolactone inactivated EV-A71 for 4 h and harvested for western blot analysis. ISG60, p-IRF3 (Ser386), STING, p-STING (Ser366) and GAPDH were probed using the indicated antibodies. GAPDH was used as a loading control. **g.** HUVEC cells were transfected with control siRNA (siNC) or siMAVS and rested for 48 h. Then, those transfected cells were mock-infected or infected with SeV at an MOI of 0.5 for 24 h and harvested for western blot analysis. MDA5, p-TBK1 (Ser172), HSP90, MAVS, ISG60, RSAD2, ISG15, and Histone H3 were probed using the indicated antibodies. HSP90 and Histone H3 were used as loading controls. **h.** HUVEC cells were transfected with control siRNA (siNC) or siMAVS and cultured for 48h. Next, cells were infected with EV-A71(MOI of 0.5) or mock-infected with a virus preservation solution. Three hours post infection, cells were harvested for western blot analysis. p-TBK1 (Ser172), MAVS, ISG60, STING, p-STING (Ser366), EV-A71 VP1, and ISG15 were probed using the indicated antibodies. HSP90 and GAPDH were used as loading controls. Data in a-e represents the average of results from three independent experiments (n = 3, representative immunoblots are shown). Error bars indicate the standard deviation of the data from three independent experiments. Means and standard deviations are presented. Statistical significance was determined using two-sided unpaired t-test, * p < 0.05; ** p < 0.01; *** p < 0.001; **** p < 0.0001.


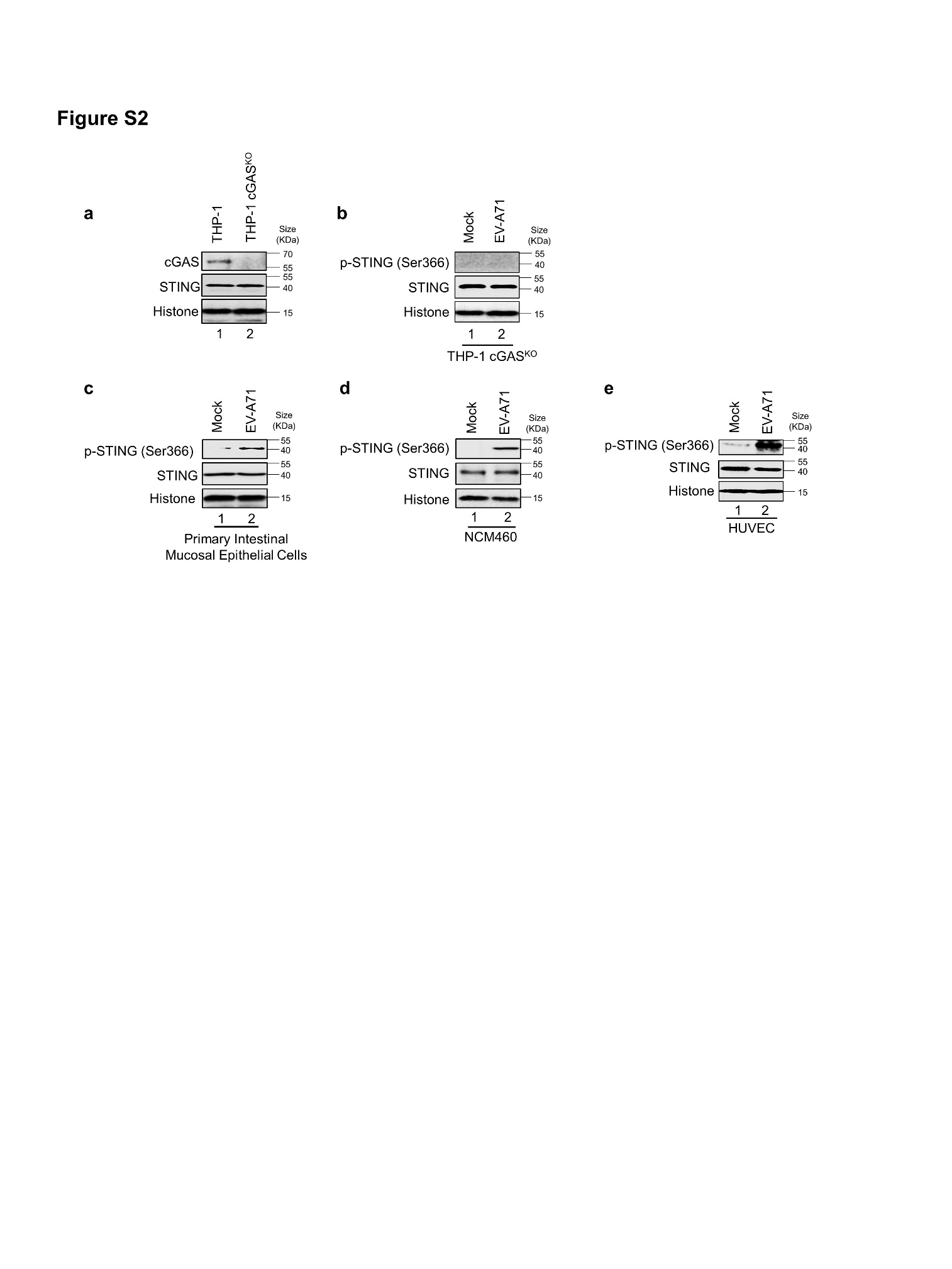


**a.** THP-1 and THP-1 cGASKO were counted and harvested with equal number of cells for western blot analysis. cGAS, STING and Histone H3 were probed using the indicated antibodies. Histone H3 was used as a loading control. **b.** EV-A71 infection failed to induce STING phosphorylation at Ser366. EV-A71 (MOI of 0.5) were used to infect THP-1 cGASKO cells (2 × 105/well in 24-well plates). Viral preservation solution was used as a negative control. Twenty-four hours later, cells were harvested for western blot analysis. STING, p-STING (Ser366), and Histone H3 were probed using the indicated antibodies. Histone H3 was used as a loading control. **c.** EV-A71 infection induced STING phosphorylation at Ser366 in human primary colonic mucosal epithelial cells. EV-A71 (MOI of 0.5) was used to infect human primary colonic mucosal epithelial cells. Viral preservation solution was used as a negative control. Two hours later, cells were harvested for western blot analysis. STING, p-STING (Ser366), and Histone H3 were probed using the indicated antibodies. Histone H3 was used as a loading control. **d.** EV-A71 infection induced STING phosphorylation at Ser366 in NCM460 cells. EV-A71 (MOI of 0.5) was used to infect NCM460 cells. Viral preservation solution was used as a negative control. Two hours later, cells were harvested for western blot analysis. STING, p-STING (Ser366) and Histone H3 were probed using the indicated antibodies. Histone H3 was used as a loading control. **e.** EV-A71 infection induced STING phosphorylation at Ser366 in HUVEC cells. EV-A71 (MOI of 0.5) was used to infect HUVEC cells. Viral preservation solution was used as a negative control. Two hours later, cells were harvested for western blot analysis. STING, p-STING (Ser366), and Histone H3 were probed using the indicated antibodies. Histone H3 was used as a loading control. Data in a-e represents the average of results from three independent experiments (n = 3, representative immunoblots are shown).


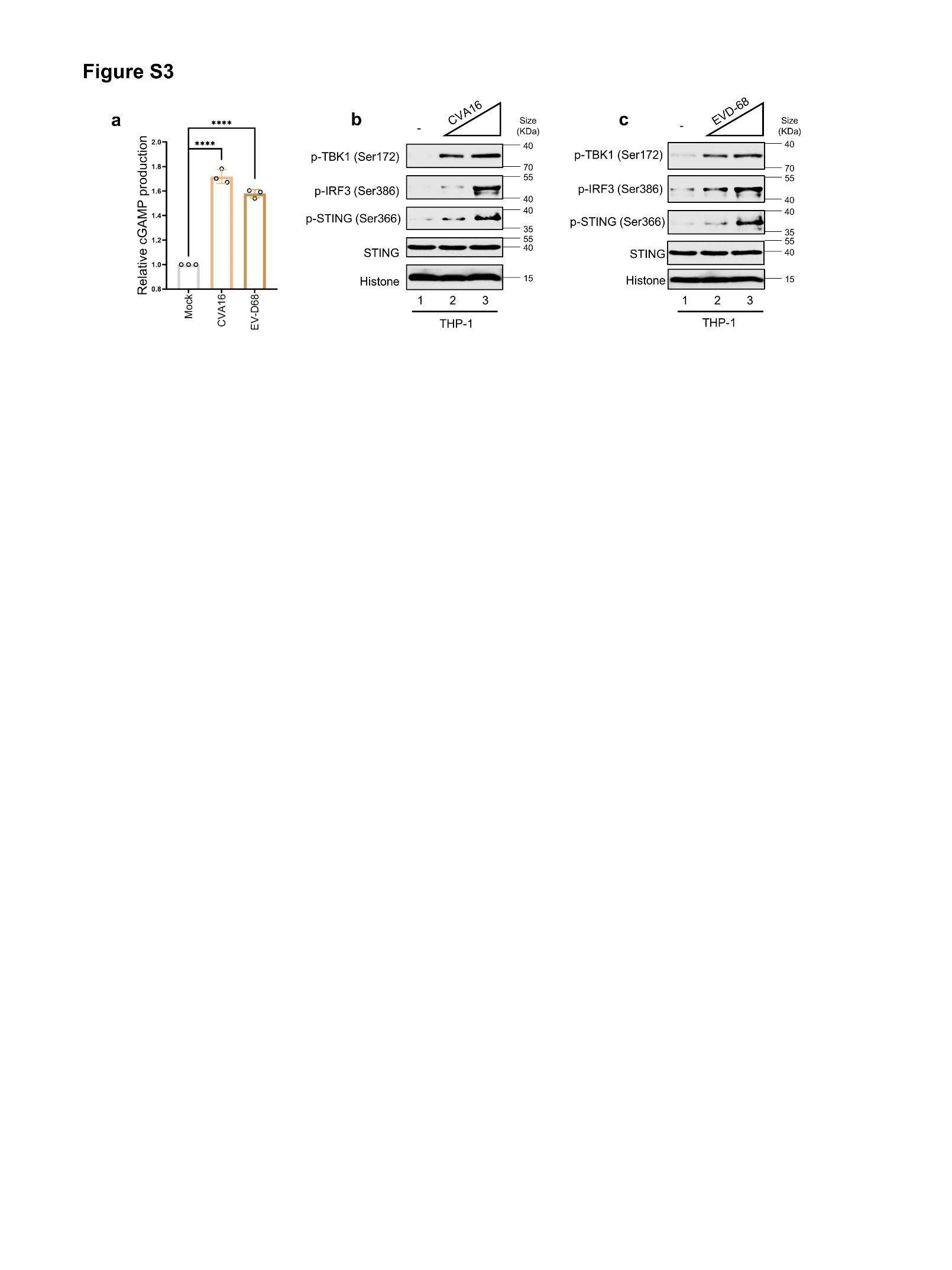


**a.** 2’3’-cGAMP levels in THP-1 cells infected with CVA16 and EV-D68 at an MOI of 0.5. THP-1 cells were infected with indicated virus for two hours and harvested for further analysis. A competitive ELISA assay determined the 2’3’-cGAMP concentrations. **b-c.** CVA16 and EV-D68 infection induce STING-triggered down-stream gene activation in THP-1 cells. EV-A71 (MOI of 0.3 and 0.6) were used to infect THP-1 cells. Viral preservation solution was used as a negative control. Two hours later, cells were harvested for western blot analysis. p-TBK1 (Ser172), p-IRF3 (Ser386), STING, p-STING (Ser366), and Histone H3 were probed using the indicated antibodies. Histone H3 was used as a loading control. Data in a-c represent the average of results from three independent experiments (n=3, representative immunoblots are shown). Error bars indicate the standard deviation of the data from three independent experiments. Means and standard deviations are presented. Statistical significance was determined using two-sided unpaired t-test, * p < 0.05; ** p < 0.01; *** p < 0.001; **** p < 0.0001.


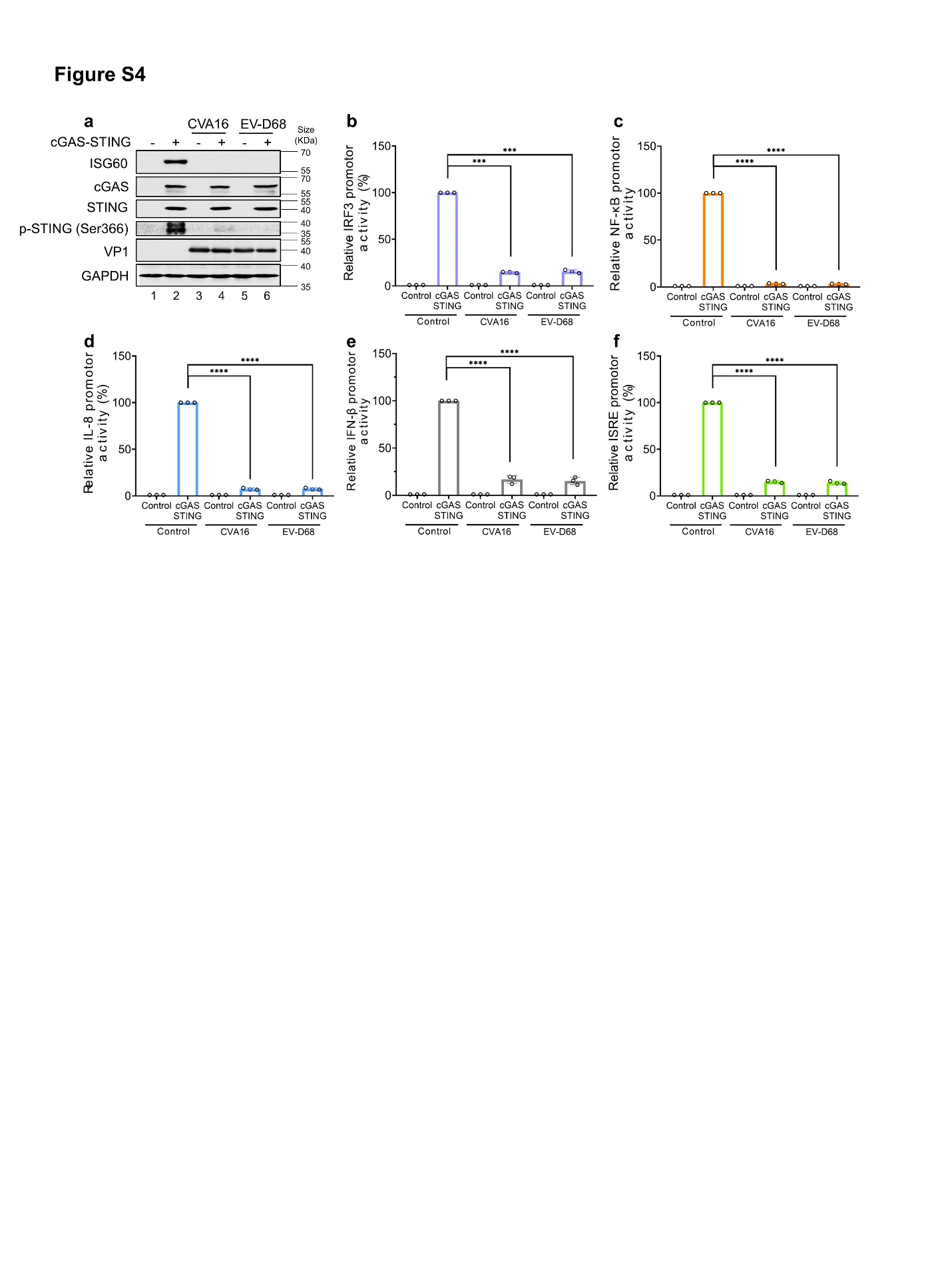


**EV-D68 and CVA16 infection inhibits cGAS-STING pathway activation.** **a.** HEK-293T cells were infected with CVA16 and EV-D68 (MOI of 0.5) or mock-infected with virus preservation solution. Twenty-four hours post infection, cells were transfected with IRF3 (**b**), NF-κB (**c**), IL-8 (**d**), IFN-β (**e**), or ISRE (**f**) promoter, together with cGAS-STING expression vectors or empty vector. Eighteen hours post-transfection, cells were harvested for western blot analysis. Cell lysates were separated by SDS-PAGE, transferred to PVDF membranes, and incubated with the indicated antibodies to detect ISG60, cGAS, STING, p-STING (Ser366), VP1, and GAPDH. GAPDH was used as a loading control. **b-f.** cGAS-STING-stimulated promoters were suppressed following EV-A71 infection. Eighteen hours post-transfection, IRF3 (**b**), NF-κB (**c**), IL-8 (**d**), IFN-β (**e**), and ISRE (**f**) promoter luciferase activity was measured using a dual-luciferase reporter gene assay. Luciferase induced by cGAS-STING served as a control and was set to 100%. pRL-TK Renilla was used as an internal control. Data in a-f represent the average of results from three independent experiments (n = 3, representative immunoblots are shown). Error bars indicate the standard deviation of the data from three independent experiments. The means and standard deviations are presented. Statistical significance was determined using a two-sided unpaired Student’s t-test, *p < 0.05; **p < 0.01; ***p < 0.001.


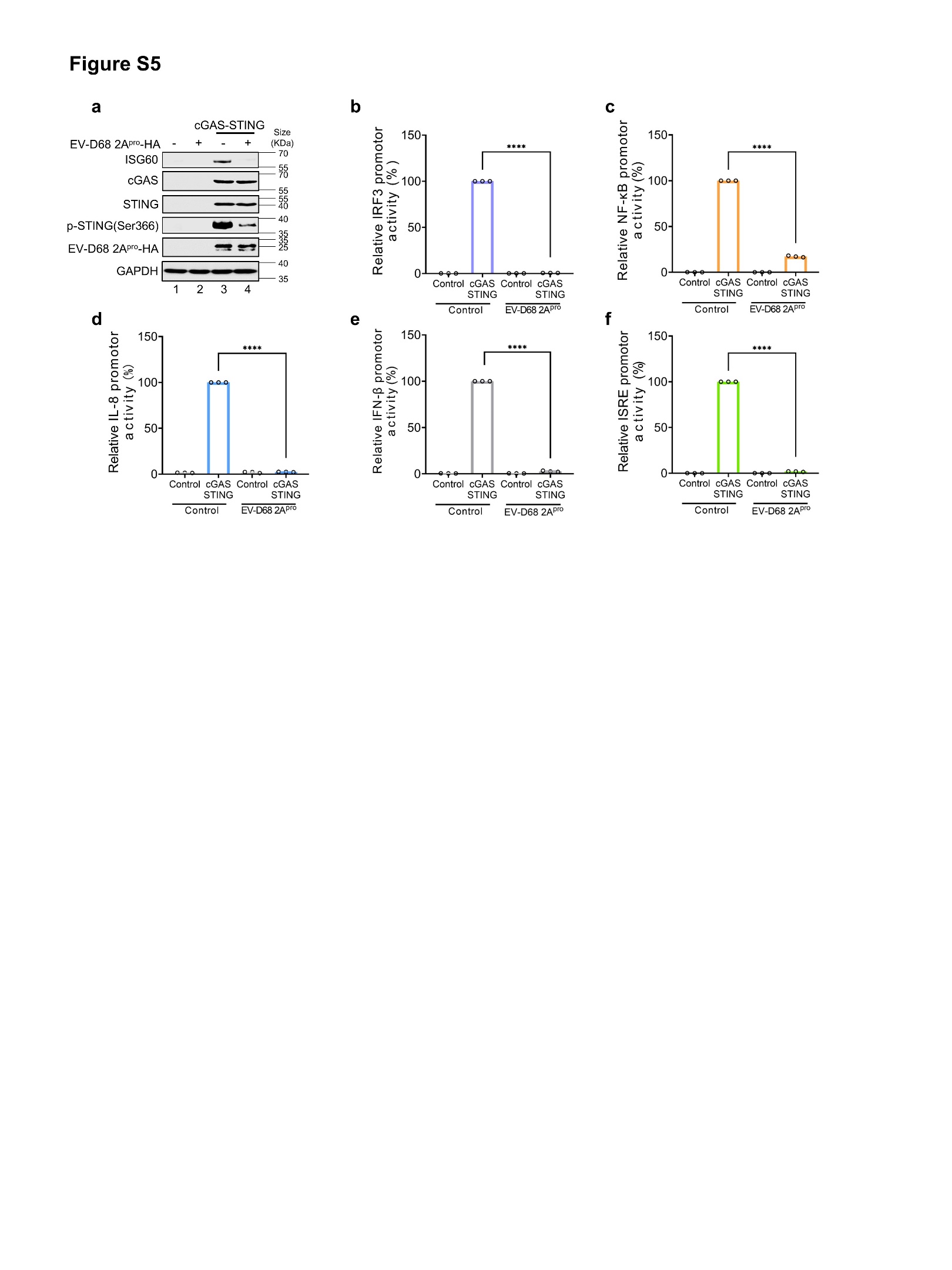


**EV-D68 2Apro inhibits cGAS-STING pathway activation. a.** EV-D68 2Apro inhibited cGAS-STING triggered STING phosphorylation and ISG60 activation. HEK-293T cells were co-transfected with empty vector, cGAS-STING-expressing vectors, empty vector, or EV-D68 2Apro expressing vector. Eighteen hours post-transfection, cells were harvested for western blot analysis. Anti-HA antibodies were used to detect EV-D68 2Apro expression. ISG60, cGAS, STING, p-STING (Ser366), and GAPDH were probed using the indicated antibodies. GAPDH was used as a loading control. b-f. EV-D68 2Apro inhibited cGAS-STING-induced IRF3, NF-κB, IL-8, IFN-β and ISRE promoter activation. HEK-293T cells were co-transfected with IRF3 (**b**), NF-κB (**c**), IL-8 (**d**), IFN-β (**e**), or ISRE (**f**) promoter, empty vector, or cGAS-STING-expressing vectors and with the presence or absence of EV-D68 2Apro expressing vectors. Eighteen hours post-transfection, cells were harvested for dual-luciferase reporter gene assays. Luciferase activity induced by cGAS-STING served as a control and was set to 100%. pRL-TK Renilla was used as an internal control. Data in a-f represent the average of results from three independent experiments (n = 3, representative immunoblots are shown). The error bars indicate the standard deviations of data from three independent experiments. Means and standard deviations are presented. Statistical significance was determined by two-sided unpaired Student’s t-test, ** p < 0.01; *** p < 0.001; **** p < 0.0001.


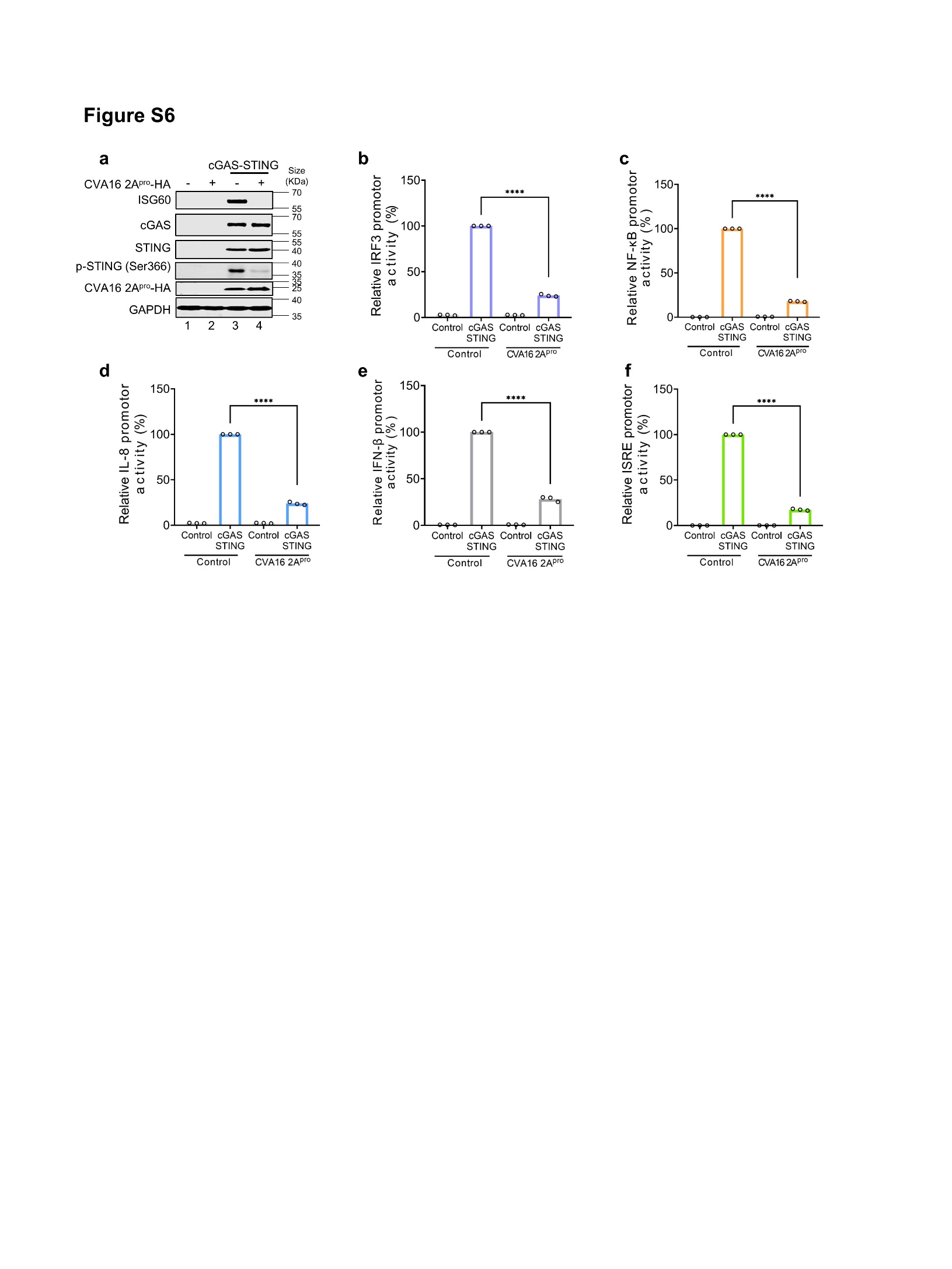


**CVA16 2Apro inhibits cGAS-STING pathway activation. a.** CVA16 2Apro inhibited cGAS-STING triggered STING phosphorylation and ISG60 activation. HEK-293T cells were co-transfected with empty vector, cGAS-STING-expressing vectors, empty vector, or CVA16 2Apro expressing vector. Eighteen hours post-transfection, cells were harvested for western blot analysis. Anti-HA antibodies were used to detect CVA16 2Apro expression. ISG60, cGAS, STING, p-STING (Ser366), and GAPDH were probed using the indicated antibodies. GAPDH was used as a loading control. b-f. CVA16 2Apro inhibited cGAS-STING-induced IRF3, NF-κB, IL-8, IFN-β, and ISRE promoter activation. HEK-293T cells were co-transfected with IRF3 (**b**), NF-κB (**c**), IL-8 (**d**), IFN-β (**e**), or ISRE (**f**) promoters’ empty vector, or cGAS-STING-expressing vectors and with the presence or absence of CVA16 2Apro expressing vectors. Eighteen hours post-transfection, cells were harvested for dual-luciferase reporter gene assays. Luciferase activity induced by cGAS-STING served as a control and was set to 100%. pRL-TK Renilla was used as an internal control. Data in a-f represent the average of results from three independent experiments (n = 3, representative immunoblots are shown). The error bars indicate the standard deviations of data from three independent experiments. Means and standard deviations are presented. Statistical significance was determined by two-sided unpaired Student’s t-test, ** p < 0.01; *** p < 0.001; **** p < 0.0001.


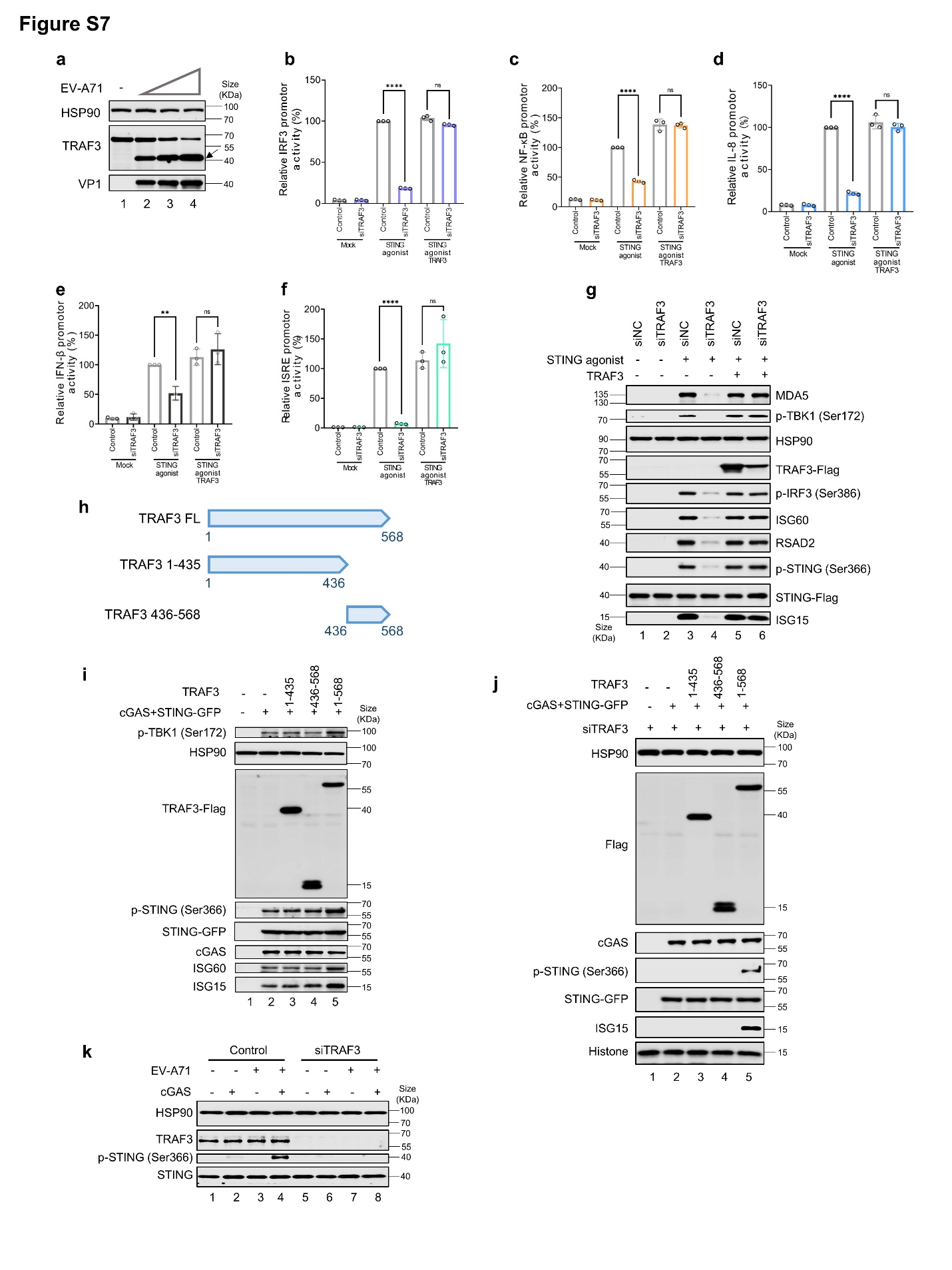


**a.** HEK-293T cells were infected with EV-A71 at an MOI of 1.0, 3.0 or 9.0 for 48h. Then, cells were harvested for western blot analysis. TRAF3, VP1 was probed with indicated antibody, GAPDH was used as a loading control. b-f. HEK-293T STING stable cell line were transfected with control siRNA (siNC) or siTRAF3. Twenty-four hours later, cells were co-transfected with IRF3 (**b**), NF-κB (**c**), IL-8 (**d**), IFNβ (**e**) or ISRE (**f**) promoters, empty vector, or TRAF3-expressing vectors for 16h. Cells were treated with STING agonist or constant volume DMSO for 8 h and then harvested for dual-luciferase reporter gene analysis. Luciferase induced by STING agonist served as a control and was set to 100%. pRL-TK Renilla was used as an internal control. Data in b-f represent the averages of three independent experiments (n = 3, representative immunoblots are shown). The error bars indicate the standard deviations of data from three independent experiments. Means and standard deviations are presented. Statistical significance was determined by two-sided unpaired Student’s t-test, ** p < 0.01; *** p < 0.001; **** p < 0.0001. **g.** HEK-293T STING stable cell line were transfected with control siRNA(si-NC) or si-TRAF3 and cultured for 36h. Next, cells were transfected with empty vectors or TRAF3 expression vectors. Sixteen hours later, cells were primed with STING agonist or constant volume DMSO for 5 h. MDA5, p-TBK1(Ser172), Flag tagged TRAF3, p-IRF3 (Ser386), ISG60, RSAD2, p-STING(Ser366), STING, and ISG15 were probed with indicated antibodies. HSP90 was used as a loading control. **h.** Expressing vectors of TRAF3 cleavage products by 2Apro, 1-435, and 436-568 were constructed. **i.** HEK-293T cells were transfected with cGAS, STING-GFP, TRAF3 (WT or truncations) expression vectors or control vectors as indicated. Sixteen-hours later, cells were harvested for western blot analysis. p-TBK1(Ser172), Flag, p-STING (Ser366), STING-GFP, cGAS, ISG60 and ISG15 were probed with indicated antibodies. HSP90 was used as a loading control. **j.** HEK-293T cells were transfected with siRNA and rested for 24h. Then, those cells were transfected with cGAS, STING-GFP, TRAF3 (WT or truncations) expressing vector or empty vector. Twenty-four hours post transfection, cells were harvested for western blot analysis. HSP90, Flag, cGAS, p-STING(Ser366), STING, ISG15 and Histone H3 were probed using the indicated antibodies. HSP90 and Histone H3 were used as loading controls. **k.** HEK-293T STING stable cell line were transfected with control siRNA (siNC) or siTRAF3 and rested for 24 h. Then, those cells were transfected with cGAS expressing vector or empty vector. Twenty-four hours post transfection, cells were mock-infected or infected with EV-A71 at an MOI of 0.5 for 4h and harvested for western blot analysis. HSP90, TRAF3, p-STING (Ser366), and STING were probed using the indicated antibodies. HSP90 was used as a loading control. Data in a-k represent the average of results from three independent experiments (n = 3, representative immunoblots are shown). The error bars indicate the standard deviations of data from three independent experiments. Means and standard deviations are presented. Statistical significance was determined by two-sided unpaired Student’s t-test, ** p < 0.01; *** p < 0.001; **** p < 0.0001.


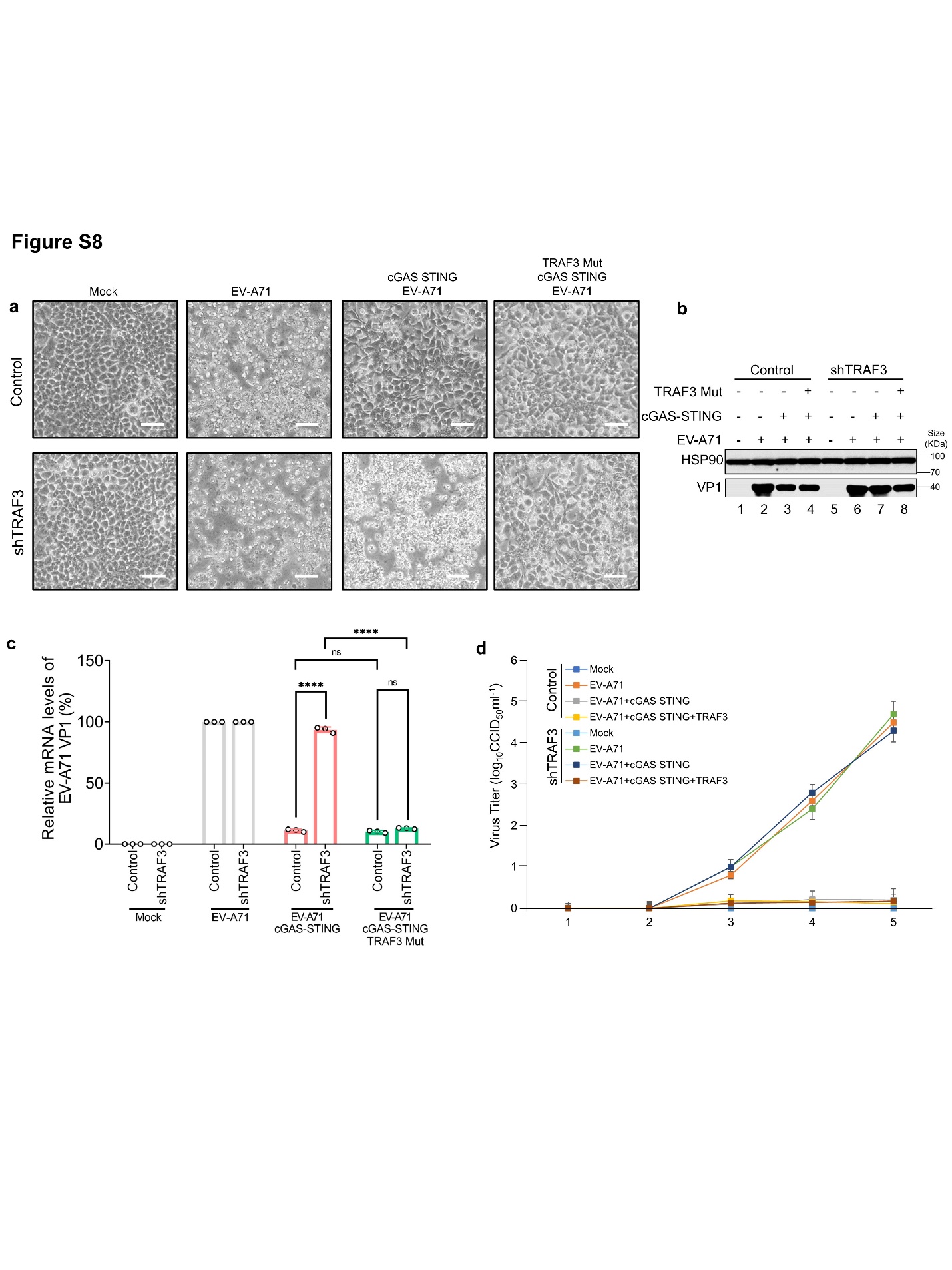


HEK-293T-Control or HEK-293T-shTRAF3 cells were co-transfected cGAS, STING and TRAF3 mutant (not cleavable by EV-A71 2Apro) expression vectors, Twelve-hours later, the cells were infected with equal amounts (MOI of 0.5) of EV-A71 for 6 h. The cells were then washed and incubated in fresh culture medium. **a.** Five days later, the cells were observed for morphological changes and photographed using light microscopy at 400 × magnification. Scale bar represents 50 μm. **b.** Seventy-two hours post infection, cells were harvested for western blot analysis. TRAF3, cGAS, STING, and VP1 were probed with indicated antibodies. HSP90 was used as a loading control. **c.** Seventy-two hours post infection, cells were harvested for total RNA extraction. The viral RNA levels of EV-A71 were evaluated using RT-qPCR with SYBR green. Primers targeted EV-A71 VP1 to monitor viral replication. GAPDH abundance was used as a control. **d.**EV-A71 from HEK-293T-control or HEK-293T-shTRAF3 cells were collected on days 1-5 after infection. The viral titer was determined using CCID50 assays. Data in a-d represent the average of results from three independent experiments (n = 3, representative cell photos and immunoblots are shown). Error bars indicate the standard deviation of the data from three independent experiments. Means and standard deviations are presented. Statistical significance was determined using two-sided unpaired t-test, * p < 0.05; ** p < 0.01; *** p < 0.001; **** p < 0.0001.
