# Supplementary material for: TRAF3 activates STING-mediated suppression of EV-A71 and target of viral evasion
Source: Signal Transduct Target Ther. 2023 Feb 24;8:79. doi: 10.1038/s41392-022-01287-2 (PMC9950063; doi:10.1038/s41392-022-01287-2)

Supplementary Materials for

TRAF3 as an activator of STING mediated suppression of EV-A71 and target of viral evasion

Wenwen Zheng^1^, Zhenbang Zhou^1^, Yajuan Rui^1^, Runxin Ye^1^, Fengyan Xia^1^, Fei Guo^3^, Xiaoman Liu^3^, Jiaming Su^1^, Meng Lou^1^, and Xiao-Fang Yu*^1,2^

1 Cancer Institute (Key Laboratory of Cancer Prevention and Intervention, China National Ministry of Education), The Second Affiliated Hospital, Zhejiang University School of Medicine, Hangzhou, Zhejiang 310009, China;

2 Cancer Center, Zhejiang University, Hangzhou, Zhejiang 310058 China

3 National Health Commission of the People’s Republic of China Key Laboratory of Systems Biology of Pathogens, Institute of Pathogen Biology and Center for AIDS Research, Chinese Academy of Medical Sciences and Peking Union Medical College, Beijing 100730, China.

Correspondence: Xiao-Fang Yu (E-mail: [xfyu1@zju.edu.cn](mailto:xfyu1@zju.edu.cn))

These authors contributed equally: Wenwen Zheng, Zhenbang Zhou, Yajuan Rui and Runxin Ye

**This PDF file includes:**

Original and uncropped films of Western blots


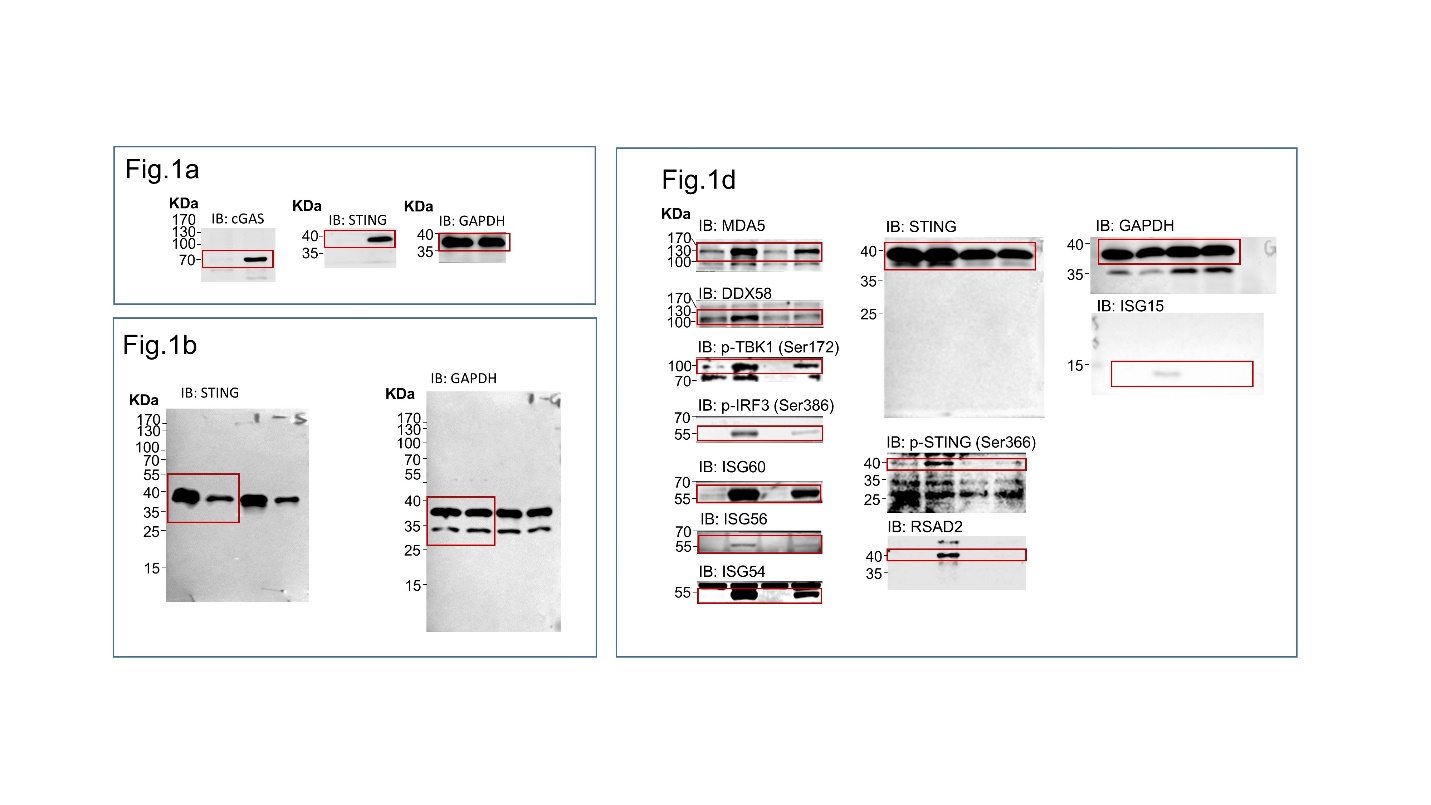


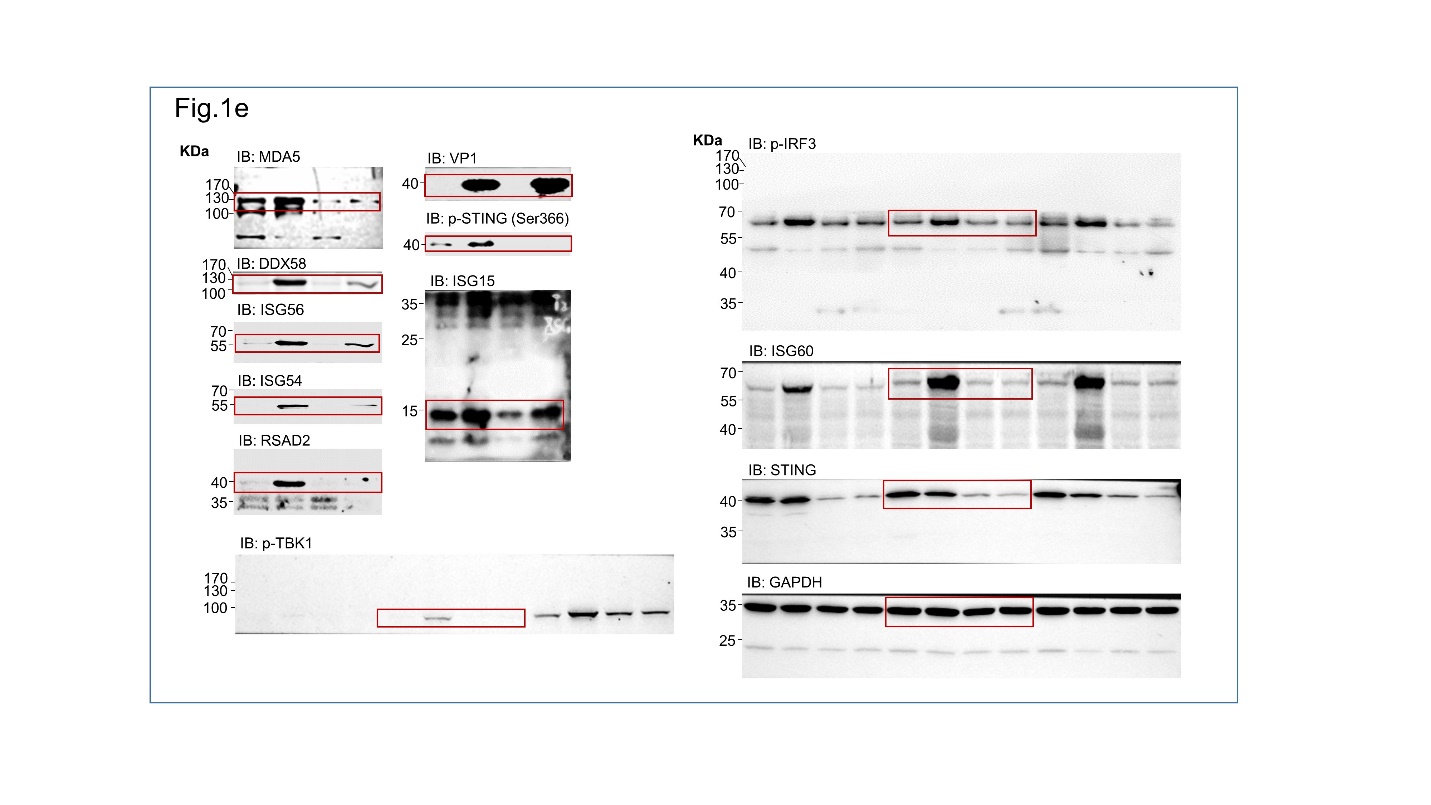


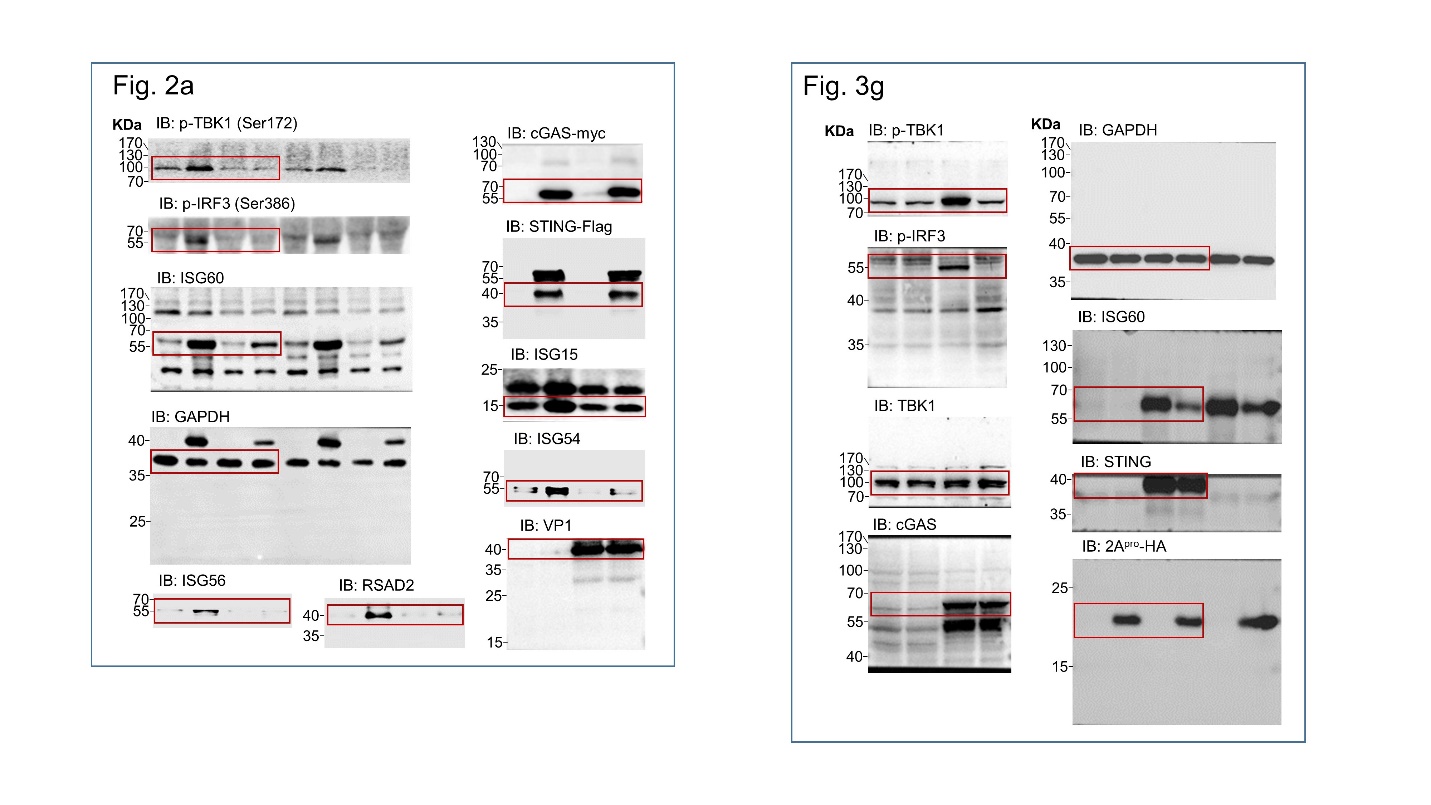


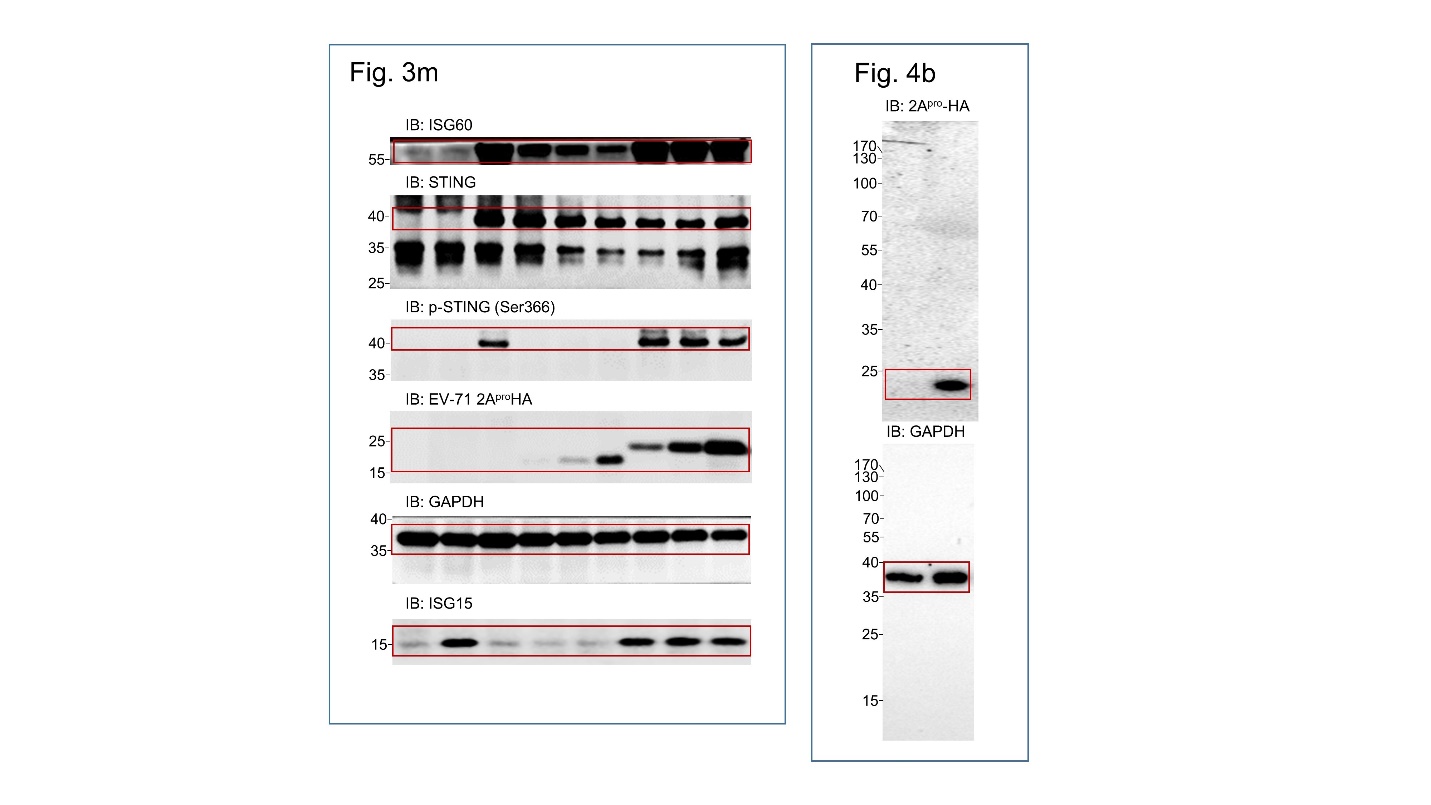


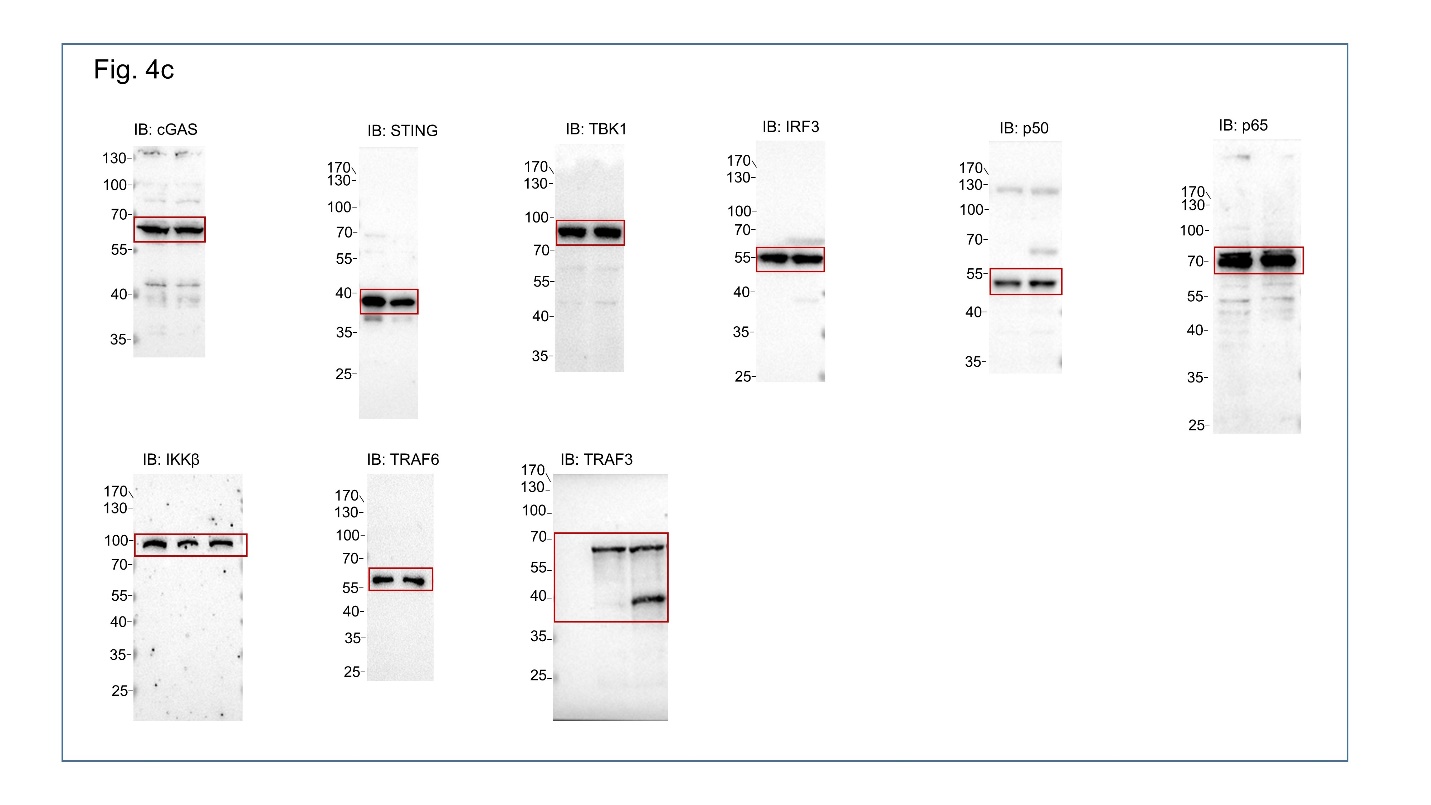


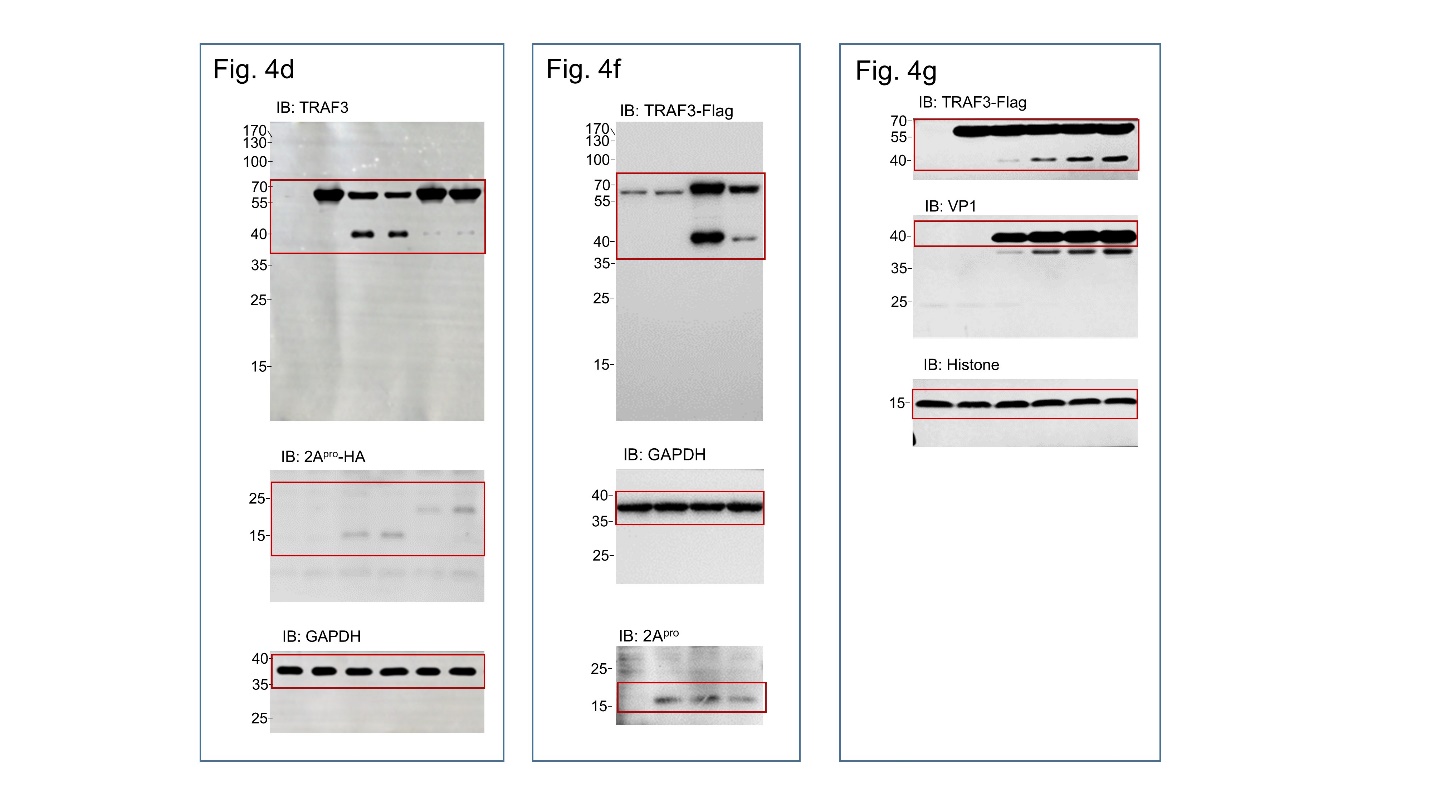


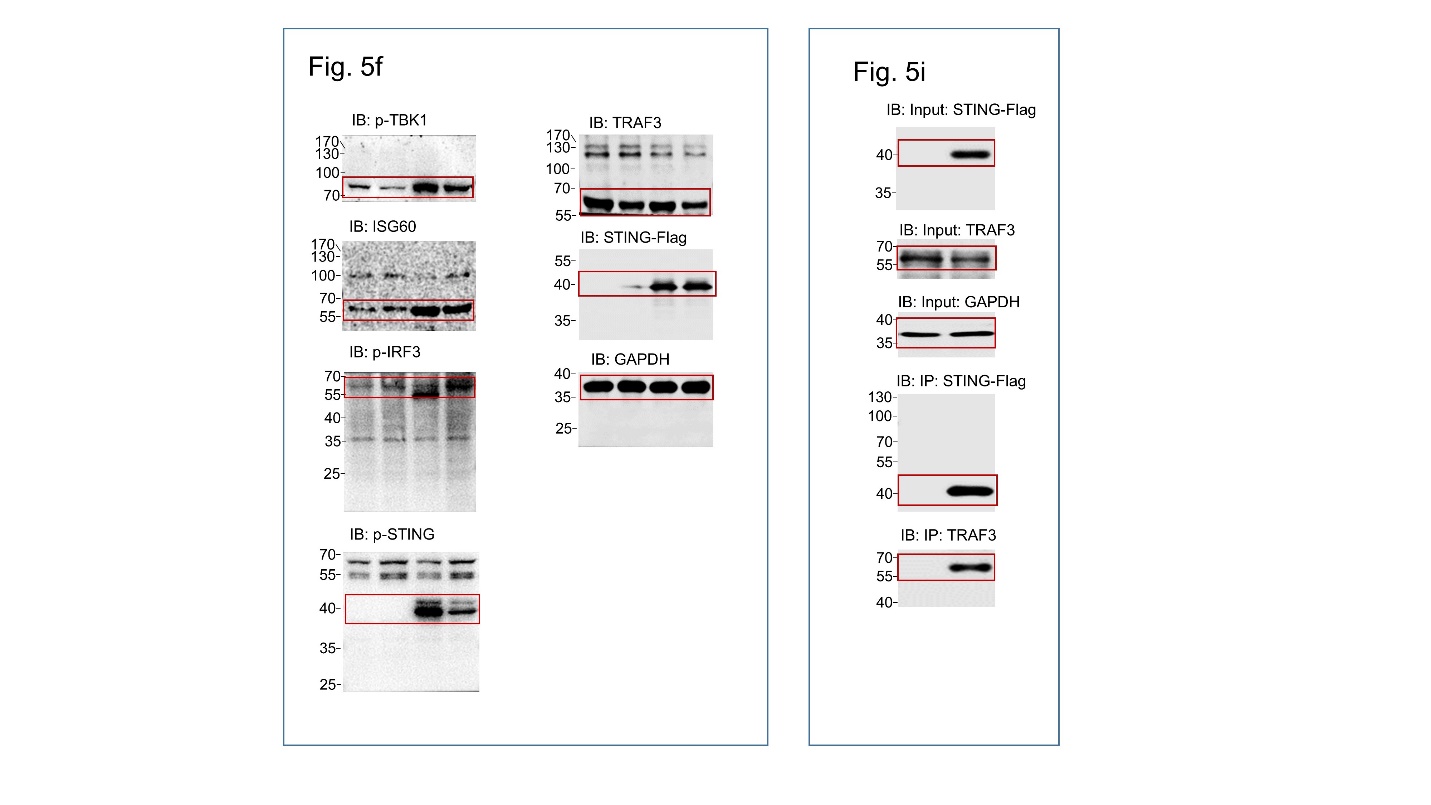


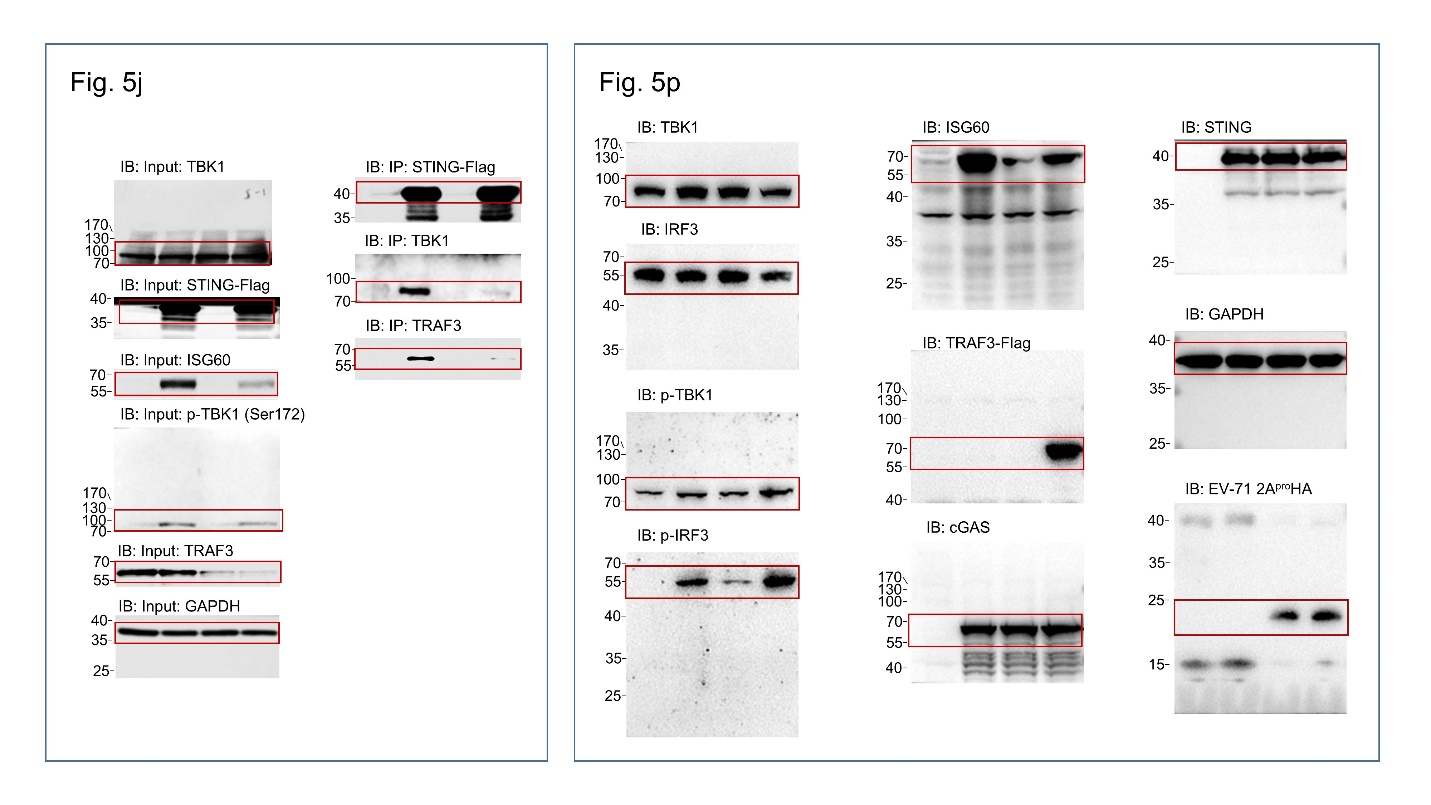


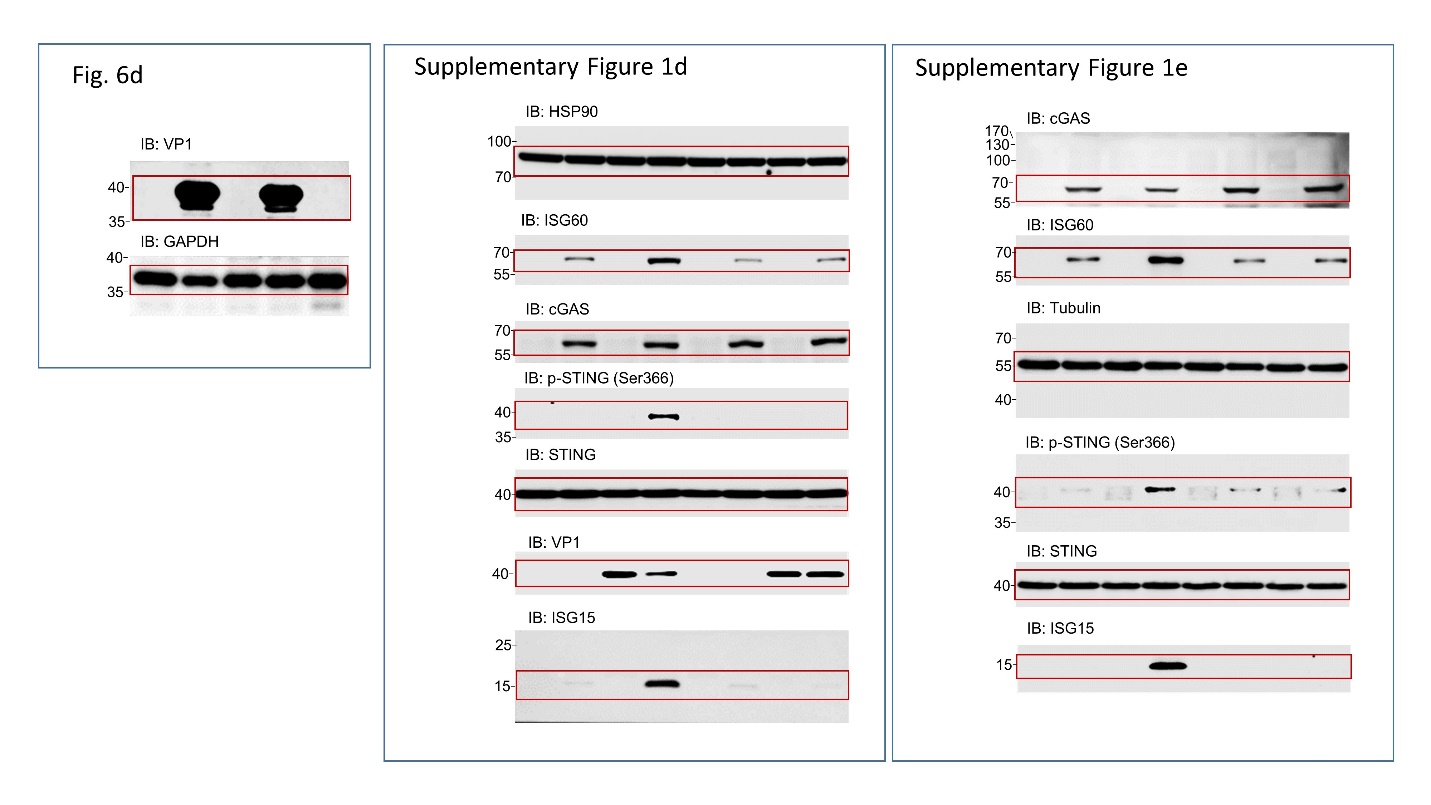


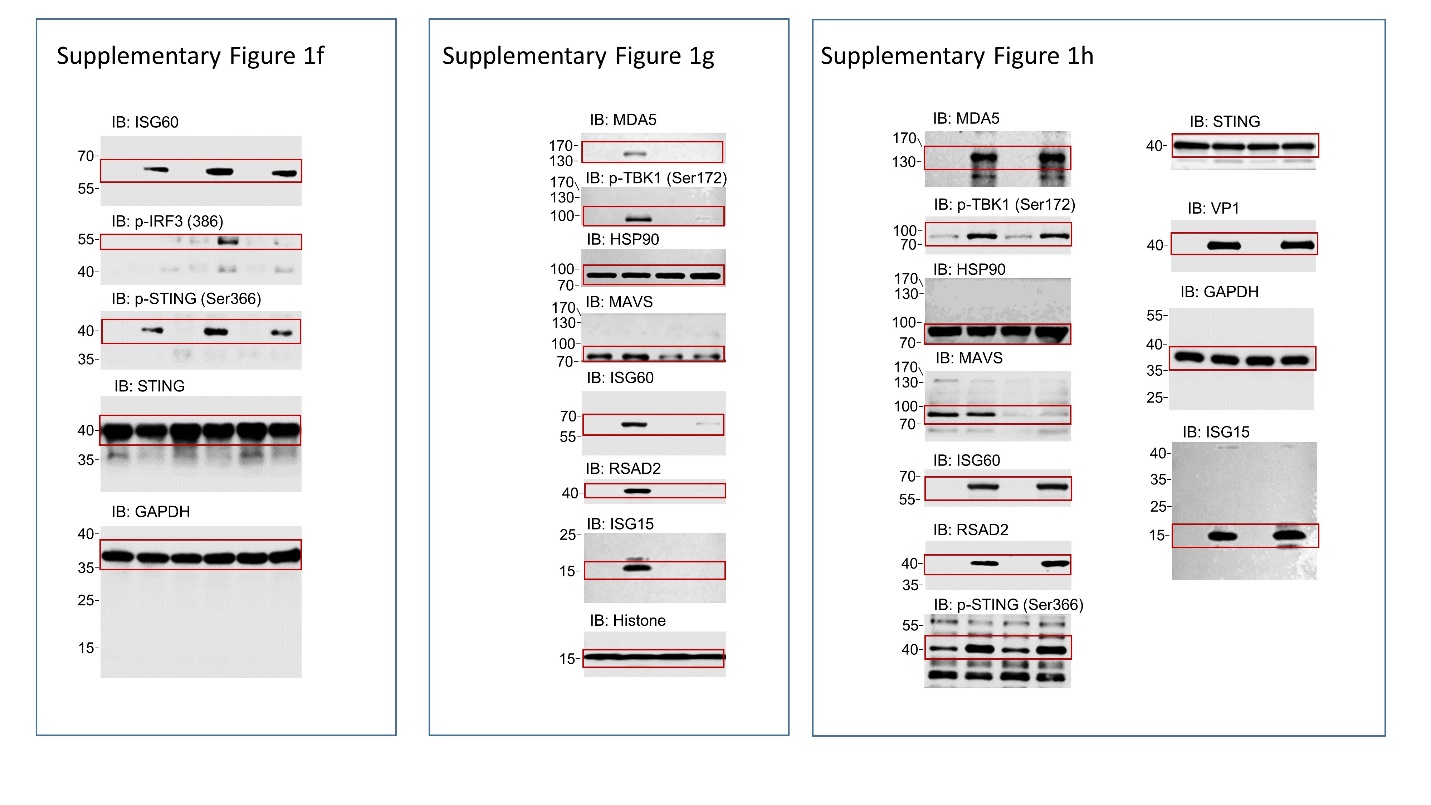


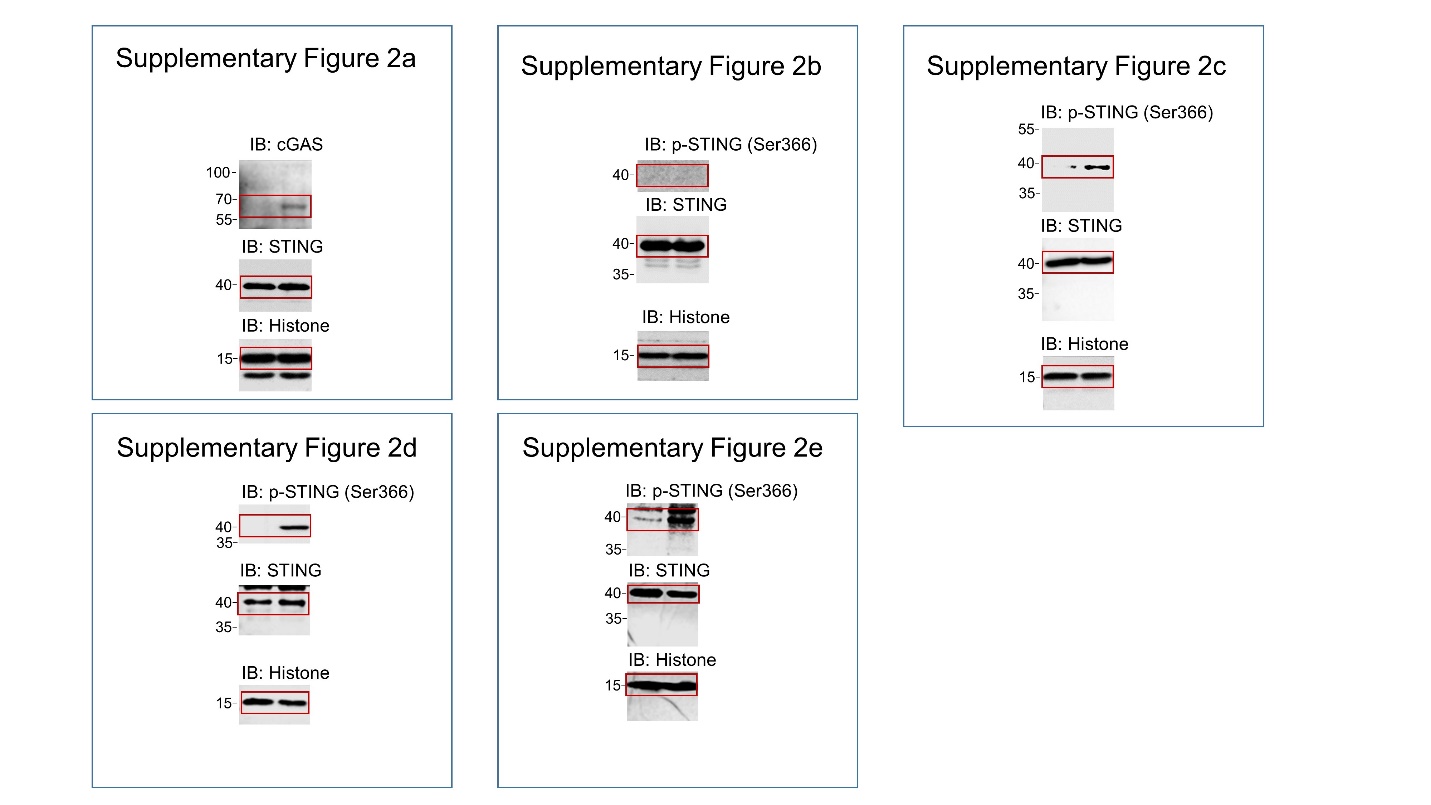


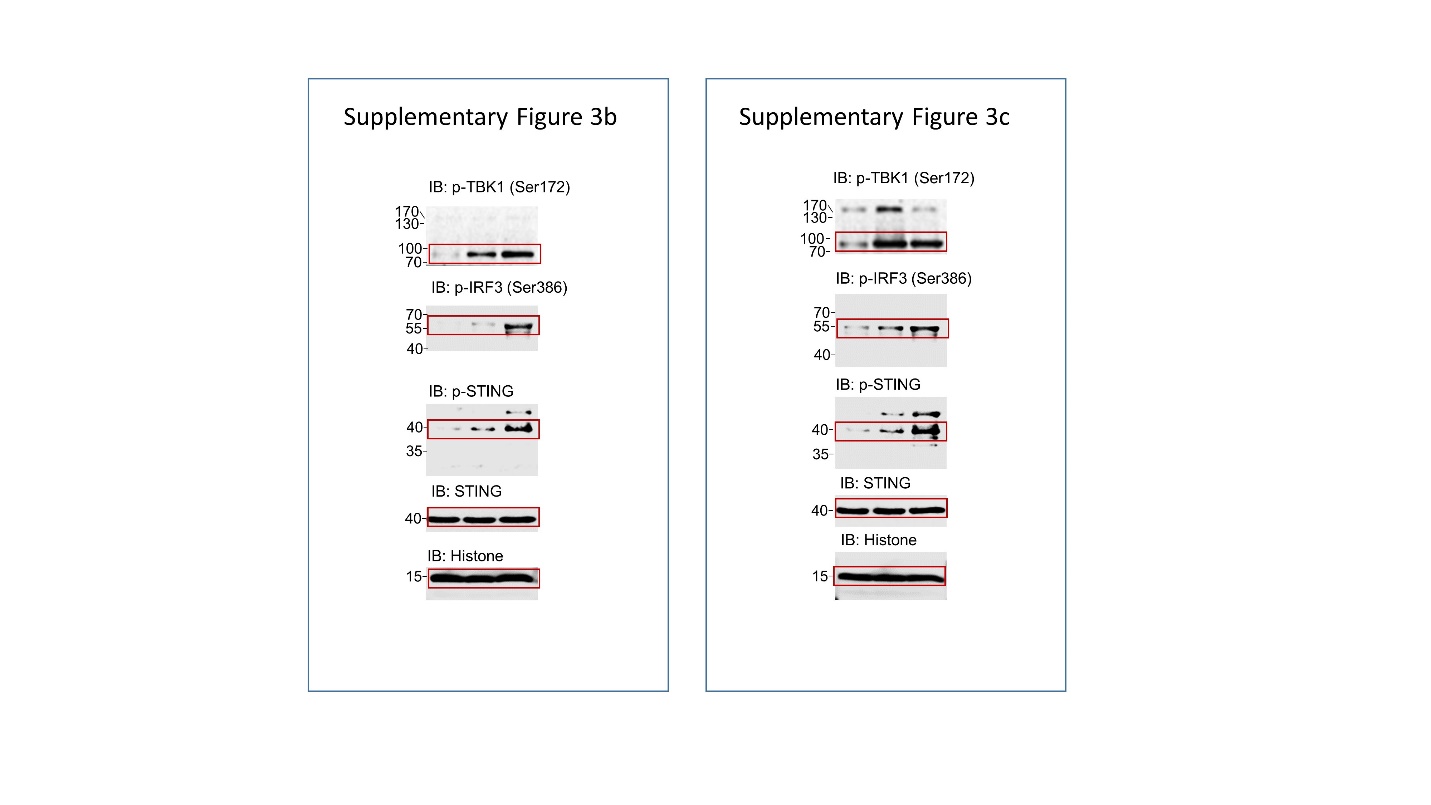


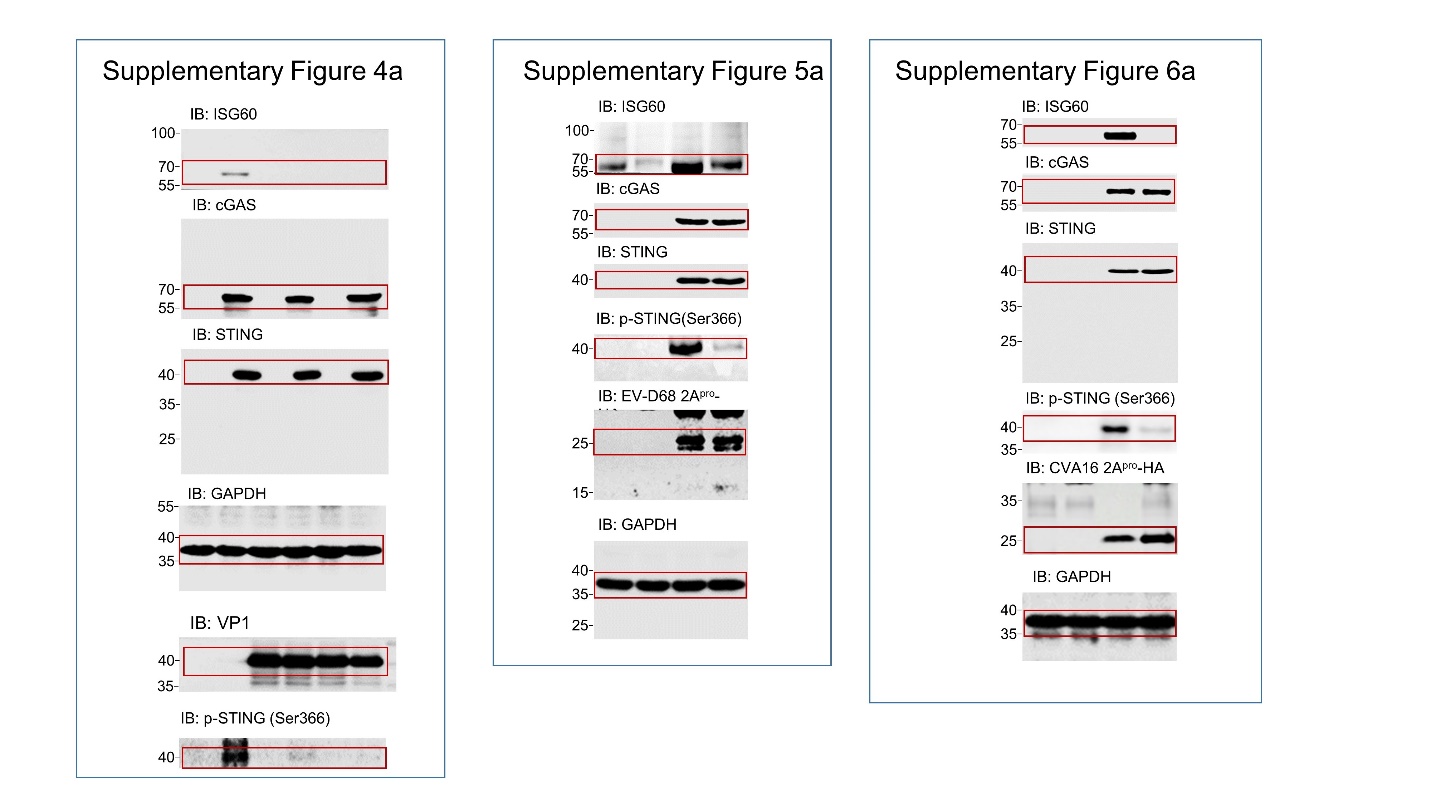


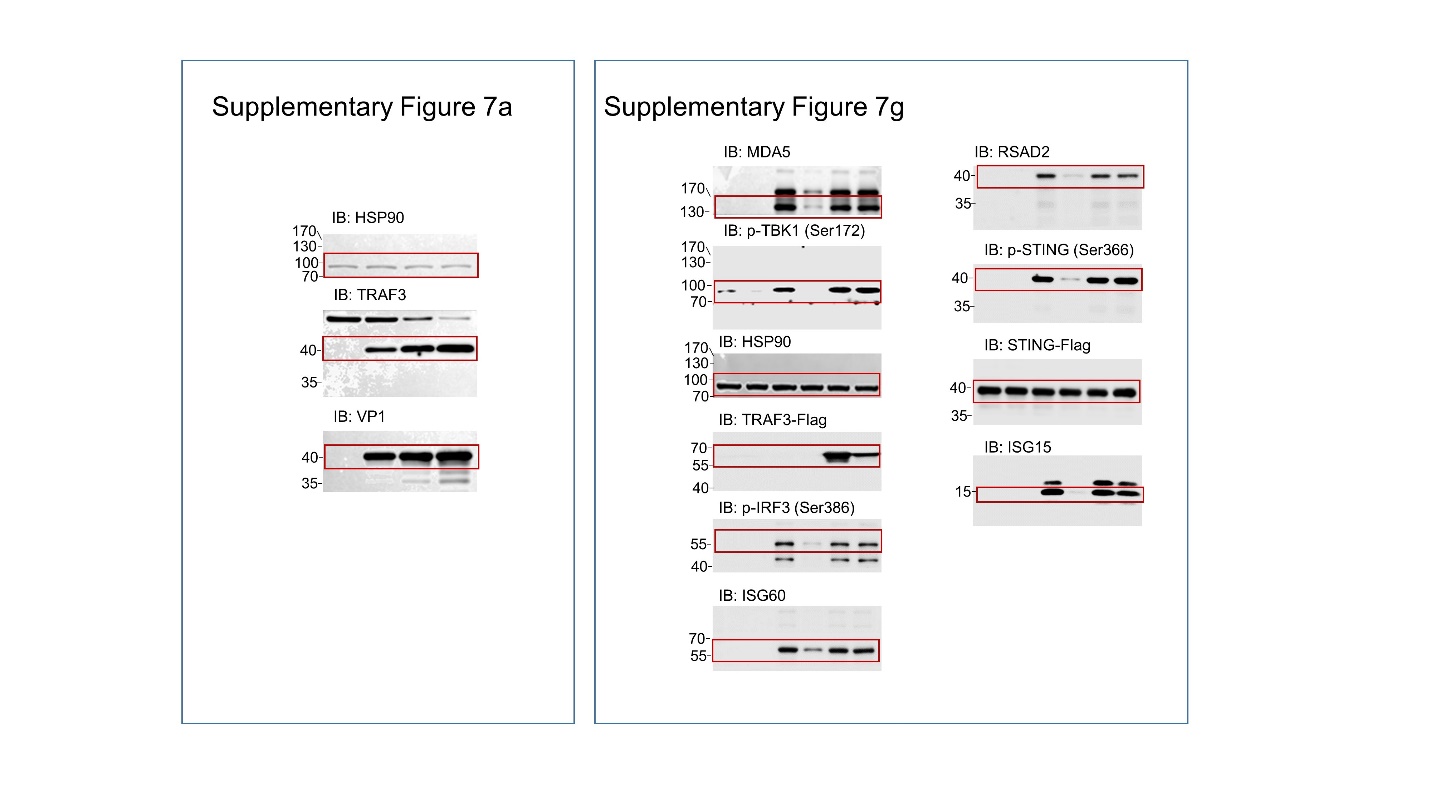


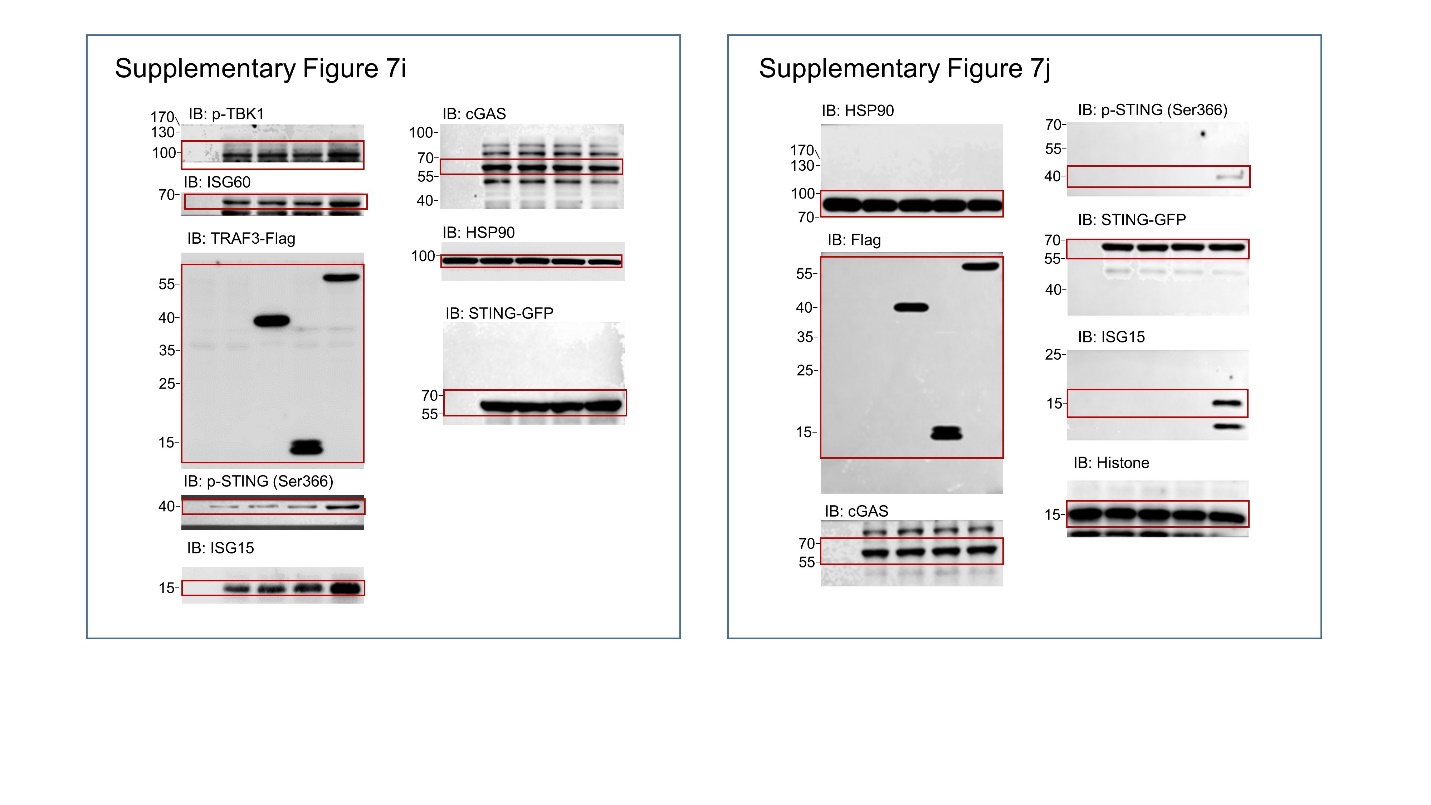


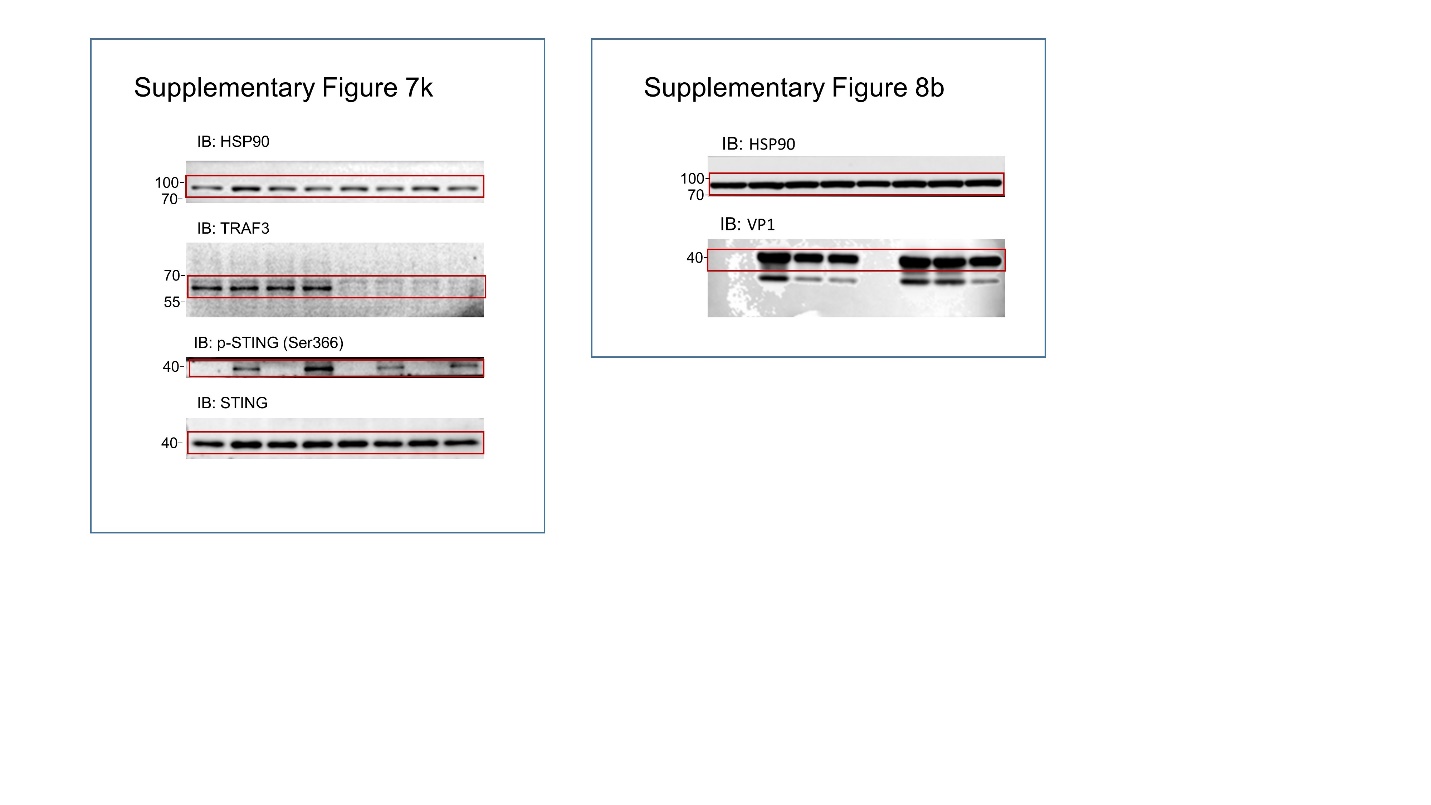

Supplement: Supplementary file 2 — Original films of Western blot [file 41392_2022_1287_MOESM2_ESM.docx]
